# Development of Replicon Cell Pools Bearing a Flavivirus RNA Replicon as a Source of HIV-1 Gag-Pol for Lentiviral Vector Production

**DOI:** 10.3390/biology15110848

**Published:** 2026-05-28

**Authors:** Aitolkyn Kydyrbayeva, Viktoriya Keyer, Tolganay Kulatay, Gulzat Zauatbayeva, Bakytkali Ingirbay, Maral Zhumabekova, Arman Abeev, Gaziza Nigmatulla, Alexandr V. Shustov

**Affiliations:** National Center for Biotechnology, Korgalzhin hwy 13/5, Astana 010000, Kazakhstan; aitolkyn.yk@gmail.com (A.K.); keer@biocenter.kz (V.K.); kulatay@biocenter.kz (T.K.); zauatbaeva@biocenter.kz (G.Z.); ingirbay@biocenter.kz (B.I.); zhumabekova@biocenter.kz (M.Z.); abeev@biocenter.kz (A.A.); nigmatullag@gmail.com (G.N.)

**Keywords:** lentiviral vectors, viral vector manufacturing, gene therapy, RNA replicons, flavivirus replicon, yellow fever virus, replicon cell pools, packaging cell lines, Gag-Pol expression

## Abstract

Lentiviral vectors (LVs) are indispensable tools in cell and gene therapy. Rising demand has created a global shortage of LVs, driving the development of novel packaging approaches. We report a novel vector packaging approach using autonomously replicating cytoplasmic RNAs (replicons) to express packaging proteins. Yellow fever virus (YFV) was used as a source of replicons encoding the HIV-1 Gag–Pol polyprotein together with reporter or selectable markers. YFV replicons were able to establish chronic infection in HEK293FT cells. Replicons expressing HIV-1 Gag–Pol containing the wild-type HIV-1 protease caused strong cytotoxicity, which prevented the selection of polyclonal cell pools harboring the replicon. In contrast, a replicon carrying the T26S mutation in the HIV-1 protease gene showed no measurable cytotoxic effects, enabling the generation of stable replicon-containing cell pools. The replicon cell pools were established using antibiotic selection and maintained Gag-Pol expression for at least ten passages under selection pressure. Using these first-generation replicon cell pools as packaging cells, LV production required only transient transfection of a transfer vector, a Tat/Rev plasmid, and an envelope plasmid. Yields reached ~10^6^ TU/mL prior to concentration and ~10^9^ TU from multilayer cell stacks, which fall within the range typically reported for conventional transient transfection systems under similar culture conditions. The resulting vectors efficiently transduced target cells, and no replication-competent lentivirus (RCL) was detected using a two-phase RCL assay with p24 ELISA detection. This demonstrator platform utilizing replicon cell pools represents a novel approach for LV packaging.

## Simple Summary

Gene and cell therapies are innovative medical approaches in which genes or living cells are used as medicines. In many cases, modified viruses known as viral vectors are used to deliver therapeutic genes. An important class of viral vectors is derived from lentiviruses and is widely used in advanced cancer treatments such as CAR-T therapy. However, large-scale production of lentiviral vectors using conventional methods remains challenging, leading to global supply limitations. To address this limitation, we developed a new method for producing lentiviral vectors using self-replicating RNA molecules derived from yellow fever virus. These RNA molecules encode the viral components required for lentiviral vector assembly. We generated cell populations that stably maintain these RNA molecules—termed replicon cell pools—and demonstrated that they can be expanded while continuously producing the required viral components. Although this is a first-generation system, it produces lentiviral vector yields within the same range as current production methods under the conditions tested. With further optimization, this approach could help address the global shortage of viral vectors and enable more affordable access to therapy.

## 1. Introduction

Gene therapy and cell-based immunotherapies have rapidly transitioned from experimental approaches to established clinical modalities. Adeno-associated virus (AAV) and lentiviral vectors (LVs) now underpin a growing number of approved therapies and ongoing clinical trials [1,2]. In particular, LVs have become a key platform for generating ex vivo genetically modified therapeutic cell products, including chimeric antigen receptor T cell (CAR-T) therapies and hematopoietic stem cell gene therapies [3,4]. Despite these advances, the expanding clinical demand for viral vectors has exposed major manufacturing constraints that limit scalability and increase production costs [5,6,7].

LVs are commonly produced by transient transfection of multiple plasmids encoding the transfer genome, HIV-1 proteins Gag–Pol, Rev and Tat, and an envelope glycoprotein [8,9]. Although robust and flexible, this approach is inherently resource-intensive and difficult to scale, contributing significantly to the high cost of LV-based therapies [10,11]. Clinical successes in diseases such as inherited hemoglobinopathies, X-linked severe combined immunodeficiency, and Wiskott–Aldrich syndrome underscore the importance of improving LV manufacturing platforms to enable broader accessibility [11,12,13]. The development of packaging cell lines (PCLs) that stably express viral components represents a promising strategy to enhance consistency and reduce cost; however, the establishment of fully stable PCLs remains technically challenging and time-consuming [8,9,14].

An alternative strategy employs non-integrating expression systems that persist in cells to generate pseudostable cell pools, such as replicon cell pools (RCPs) harboring cytoplasmically replicating viral replicons. Viral replicons are self-replicating RNA molecules derived from positive-sense RNA viruses in which structural protein genes are removed, preventing infectious particle formation while retaining autonomous cytoplasmic replication [15,16]. Because replicons achieve high intracellular copy numbers independently of nuclear transcription, they can sustain robust protein expression without genomic integration. In contrast, the generation of fully stable lentiviral producer cell lines typically require genomic integration of multiple viral components followed by extensive clone screening and optimization, which can be technically challenging and time-consuming [17,18]. RCPs based on cytoplasmically replicating RNAs occupy an intermediate niche between transient transfection and stable cell lines, eliminating the tedious clonal selection step and thereby shortening the development of expressing cell pools [16,19]. Moreover, they preserve expression longer than plasmid transfection because self-amplifying RNA can provide durable, non-integrative transgene expression [20,21]. This principle is exemplified by technologies that offer the same developmental speed advantages as published episomal systems, such as EBV replicon vectors [22] or QMCF technology [23,24].

Replicons from positive-sense RNA viruses are widely used as self-amplifying RNAs due to their high replication efficiency and ease of genetic manipulation. However, certain RNA(+) viruses have been reported to exhibit a propensity for RNA recombination between co-replicating molecules via copy-choice mechanisms [25,26,27]. Such recombination could generate replication-competent lentivirus (RCL), posing a hypothetical safety concern for therapeutic applications [28,29]. However, second-generation (three-plasmid) packaging systems already require 2–3 simultaneous recombination events to generate RCL [28]. Accordingly, contemporary second-generation systems are associated with extremely low RCL incidence [29]. Notably, the probability of generating a single RCL genome molecule at production scale is estimated at ≤1 in 10,000 per 200 L bioreactor [28].

Unlike recombination-prone RNA viruses, certain positive-sense RNA viruses exhibit naturally low recombination frequencies due to their specialized replication strategies. Their RNA synthesis occurs within highly organized intracellular replication compartments that restrict interactions between co-replicating RNA molecules. For example, consistent with this organization, flaviviruses (genus *Orthoflavivirus*, family *Flaviviridae*) display intrinsically low recombination rates, as reflected by the rarity of viable recombinants both in nature and under experimental conditions designed to promote recombination [30,31]. Flavivirus replicons engineered to express HIV proteins may therefore provide a safer platform for LV packaging when minimizing RCL risk is a critical safety requirement.

One challenge in developing constitutive LV packaging systems is the cytotoxicity associated with HIV-1 protease (PR) activity. HIV-1 PR cleaves host cellular proteins, and its expression alone has been shown to be intrinsically toxic in mammalian and bacterial cells [32]. This toxicity complicates the development of cell lines continuously expressing the Gag–Pol polyprotein. Mutations that attenuate protease activity while preserving sufficient function for virion maturation offer a potential solution.

In this study, we describe a novel LV packaging strategy based on a replicon derived from the model flavivirus yellow fever virus (YFV). We engineered bicistronic YFV replicons encoding HIV-1 Gag–Pol, either wild-type or carrying the attenuating T26S protease mutation, together with selectable or reporter markers. We evaluated replicon cytotoxicity and the feasibility of establishing RCP capable of sustained replicon replication and continuous Gag–Pol expression. Furthermore, we optimized LV production using this platform and assessed its scalability.

The flavivirus replicon–based system described here represents a new direction in packaging platform development and may enable more cost-effective LV manufacturing while maintaining a favorable biosafety profile.

## 2. Materials and Methods

### 2.1. Cell Cultures

HEK293FT cells (Thermo Fisher Scientific, Cat. R70007, Waltham, MA, USA) were cultured in Dulbecco‘s Modified Eagle’s Medium (DMEM) with high glucose (Gibco Cat. 11965092), supplemented with 10% fetal bovine serum (FBS; Gibco Cat. 10091148, Grand Island, NY, USA), 1% penicillin-streptomycin (Pen/Strep), 1% non-essential amino acids (NEAA), and 2 mM L-glutamine (all from Gibco, Grand Island, NY, USA). Cells were maintained at 37 °C in a 5% CO_2_ atmosphere. To produce replicon cell pools (RCPs), the culture medium was supplemented with 10 µg/mL puromycin (Sigma-Aldrich Cat. P8833, St. Louis, MO, USA). C8166 cells (Merck Cat. 88051601, Darmstadt, Germany) were maintained as suspension cultures in RPMI-1640 medium supplemented with 10% fetal bovine serum (FBS), 1% penicillin-streptomycin, 1% NEAA, and 2 mM L-glutamine. C8166 cells were seeded at a density of 1 × 10^5^ cells/mL in T25 flasks and subcultured when the density reached 1 × 10^6^ cells/mL.

### 2.2. Construction of Replicons and Molecular Infectious Clones

The replicons were derived from the yellow fever virus (YFV) vaccine strain 17D. A replicon is a viral genome fragment lacking structural protein genes but retaining essential cis-acting sequences (5′ and 3′ UTRs, and a cyclization signal) and the replicase genes, enabling autonomous cytoplasmic replication.

The YFV replicons retain the essential cyclization signal, which is present within a fragment of the capsid protein gene encoding the first 25 amino acids (a.a.) of the C protein, thereby preserving replication capacity. The YFV structural protein genes (C-prM-E) were replaced with a bicistronic gene cassette which consists of HIV-1 Gag-Pol genes (with a stop codon), the internal ribosome entry site of encephalomyocarditis virus (EMCV IRES), and a gene for an additional heterologous protein to simplify vector titration (GFP) or enable selection (puromycin acetyltransferase, Pac). The GAG-POL genes were amplified from psPAX2 (Addgene Cat. 12260, Watertown, MA, USA). The POL reading frame terminates with a stop codon, so to ensure translation of the proteins encoded by the genes downstream of POL, the EMCV IRES was used, thus creating a bicistronic construct. The EMCV IRES was amplified from the plasmid R5 (Addgene Cat. 51733, Watertown, MA, USA). Self-cleaving peptides (*Thosea asigna* T2A, or foot-and-mouth disease virus F2A) are introduced to separate YFV and heterologous proteins to ensure their release as discrete polypeptides.

The replicon YFrep/GFP-GAG-POL contains a fragment of the C gene encoding the first 25 a.a., followed by a GFP gene fused to the F2A self-cleaving peptide and the HIV-1 GAG-POL genes; downstream of the native POL stop codon, an EMCV IRES drives translation from an engineered start codon into the C-terminal 23 a.a. of the YFV E protein, which serves as a signal peptide for NS1 translocation and thereby directs translation of the non-structural proteins NS1–NS5. The replicon YFrep/GFP-GAG-POL* is identical to YFrep/GFP-GAG-POL except that it carries mutations in the HIV-1 protease (PR) coding sequence, changing the 26th amino acid from threonine to serine (T26S).

The replicon YFrep/GAG-POL/Pac contains a fragment of the C gene encoding the first 25 a.a., followed by a sequence encoding the T2A self-cleaving peptide and the HIV-1 GAG-POL genes; downstream of the native POL stop codon, an EMCV IRES drives translation from an engineered start codon into the Pac gene, which is fused to an F2A self-cleaving peptide and then to a signal peptide for NS1 translocation, followed by the non-structural proteins NS1–NS5. The replicon YFrep/GAG-POL*/Pac is identical to YFrep/GAG-POL/Pac except that it carries a mutated sequence encoding the HIV-1 protease (T26S mutant).

Replicons were constructed as DNA-launched molecular infectious clones (MICs). In these plasmids, the replicon sequences are cloned downstream of the human cytomegalovirus (CMV) immediate-early promoter, while the hepatitis delta virus antigenomic ribozyme (HDV-Rz) followed by the human growth hormone (HGH) polyadenylation signal is placed downstream of the viral 3′ terminus to ensure proper RNA processing. RNA replicons here are designated without a “p” prefix (e.g., YFrep/GFP-GAG-POL), whereas the corresponding MIC plasmids carry a lowercase “p” prefix (e.g., pYFrep/GFP-GAG-POL). Plasmids were isolated by alkaline lysis and purified for transfection by banding in cesium chloride density gradients as described in [33].

All cloning was performed using restriction enzyme digestion and T4 DNA ligase-mediated ligation (enzymes from New England Biolabs) according to the manufacturer’s protocols. Ligated products were transformed into chemically competent *E. coli* DH5α cells, and positive clones were verified by Sanger sequencing. The detailed molecular organization of the replicons is presented in Supplementary Material Figures S1 and S2. The complete nucleotide sequence of the key construct in this study—the molecular infectious clone used to produce RCP cells (pYFrep/GAG-POL*/Pac)—is shown in Supplementary Material Figure S3. Other sequences are available from the authors upon request.

### 2.3. Construction of Transfer Vectors, Transactivator, and Envelope Plasmids

Transfer vectors LV/CAR and LV/CAR-GFP were described in the work [34]. These are second-generation self-inactivating LVs designed to deliver the gene encoding a chimeric antigen receptor (CAR). The CAR used in this study (CAR.TM8-BBz) has the same domain organization as the publicly reported CAR used in Kymriah therapy [35]. CAR expression is driven by the human EF-1α promoter. LV/CAR encodes CAR alone, while LV/CAR-GFP encodes CAR fused to GFP via a non-cleavable linker, to facilitate monitoring of transfection efficiency and vector titration.

The transactivator plasmid pTat/Rev was constructed for this study to provide the essential HIV-1 regulatory proteins Tat and Rev. Tat is required for transactivation of the LTR promoter in second-generation LVs, while Rev mediates the nuclear export of unspliced vector genomic RNA. The Tat gene sequence was derived from the pNL4-3 molecular clone (GenBank Acc. AF324493). The Rev gene was amplified from plasmid pRSV-Rev (Addgene Cat. 12253). To construct pTat/Rev, the pMD2.G plasmid (Addgene Cat. 12259) was used as the initial backbone. The VSV-G gene was removed, and the Tat gene was placed under the control of the CMV promoter, downstream of the β-globin intron present in pMD2.G. The genes encoding Tat and Rev were placed in separate cistrons linked by an EMCV IRES (from Addgene Cat. 51733). Transcription is terminated by the β-globin poly(A) signal present in the initial plasmid.

The envelope plasmid pMD2.G (Addgene Cat. 12259) encodes the envelope protein G of the vesicular stomatitis virus (VSV-G) under the control of the CMV promoter.

### 2.4. Assessment of Cytotoxicity of Gag-Pol and GFP-Expressing Replicons

The cytopathic effect (CPE) of replicon replication was evaluated following transfection of HEK293FT cells with YFrep/GFP-GAG-POL (expressing wild-type protease) or YFrep/GFP-GAG-POL* (expressing T26S mutant protease). Mock-transfected control cultures were electroporated with PBS in the absence of plasmids. Immediately after electroporation, the cells were counted and seeded into four 96-well plates at 10,000 cells per well. At 24 h post-transfection, one plate was removed from the incubator, and cell viability was measured using a 3-(4,5-dimethylthiazol-2-yl)-2,5-diphenyltetrazolium bromide (MTT) assay as described in [36]. Cell viability measurements were performed with the remaining plates at 48, 72, and 96 h post-transfection. This experiment was performed in three biological replicates.

### 2.5. Western Blot

To analyze the expression and processing of HIV-1 Gag, HEK293FT cells were transfected by electroporation with pYFV/GFP-GAG-POL or pYFV/GFP-GAG-POL*. Cells were harvested 48 h post-transfection, washed with ice-cold PBS, and lysed in 2× Laemmli sample buffer. The lysates were then boiled for 3 min. Proteins were resolved by SDS-PAGE on a 10% polyacrylamide gel and transferred onto a nitrocellulose membrane (Protran, Merck, Cat. 10600004, Darmstadt, Germany). The membrane was blocked in PBS-T (PBS with 0.1% Tween-20) containing 5% non-fat dry milk. For immunodetection, the membrane was incubated with a primary mouse monoclonal anti-HIV-1 p24 antibody (dilution 1:1000; Abcam Cat. ab9071, Cambridge, UK), followed by an IRDye 680RD-conjugated donkey anti-mouse IgG secondary antibody (dilution 1:20,000; LICORbio Cat. 926-68072, Lincoln, NE, USA). The membrane was scanned using an Odyssey DLx infrared imaging system (LI-COR Biosciences, Lincoln, NE, USA). Recombinant p24 protein (Thermo Scientific Cat. RP-4916, Waltham, MA, USA) was used as a positive control.

### 2.6. Establishment of a Replicon Cell Pool

HEK293FT cells were grown to ~90% confluence in P150 dishes, harvested and used for electroporation. Cells (2 × 10^7^) in PBS (HyClone Cat. SH30256, Logan, UT, USA) were mixed with 10 µg of plasmid (MIC) DNA in a 2 mm gap cuvette and electroporated using a Gene Pulser II apparatus (Bio-Rad, Hercules, CA, USA) with two exponential-decay pulses (1500 V, 25 µF, infinite resistance). Cells were recovered for 10 min at room temperature and transferred to P100 dishes in complete medium. When the culture reached 90% confluence, the cells were reseeded into larger dishes.

At 48 h post-transfection, the culture medium was replaced with fresh medium containing 10 µg/mL puromycin. The survived HEK293FT-GP cells represent a polyclonal population of puromycin-resistant cells, which were expanded, cryopreserved and used to package LVs.

During these experiments, the production of virus-like particles (VLPs) in culture supernatants was monitored semi-quantitatively using the Lenti-X GoStix Plus system (Takara Bio Cat. No. 631280, San Jose, CA, USA), as this served as an indicator of Gag-Pol expression.

### 2.7. Small-Scale Lentiviral Vector Production Using Replicon Cell Pools (RCPs)

HEK293FT-GP cells were seeded in 10 cm dishes at a density of 1 × 10^5^ cells/cm^2^. One hour prior to transfection, the medium was replaced with 10 mL of serum- and antibiotic-free DMEM. Cells were co-transfected with a total of 45 µg of plasmid DNA (molar ratio 2:1:1 for the transfer vector LV/CAR-GFP, transactivator pTat/Rev, and envelope pMD2.G) via the calcium phosphate precipitation (CaPi) method [37]. The plasmid DNA was brought to a final volume of 900 µL with 0.25 M CaCl_2_ solution. The resulting mixture was added dropwise to 900 µL of 2× HeBS buffer (pH 7.12; 274 mM NaCl, 10 mM KCl, 1.4 mM Na_2_HPO_4_, 15 mM glucose, 42 mM HEPES) under continuous vortexing. After a 5 min incubation at room temperature, the 1.8 mL of CaPi precipitates were added to cells and distributed in the medium. Fetal bovine serum (FBS) was added to a final concentration of 10% one hour after CaPi addition. The medium was replaced with fresh complete medium 12 h later, marking the end of the transfection procedure (zero hours post transfection). Supernatants containing LV particles were harvested at 48 h post-transfection, clarified by centrifugation (1000× *g*, 10 min) and filtered through a 0.45 μm polyethersulfone (PES) filter.

### 2.8. p24 ELISA

The production of p24-containing particles by cell cultures engineered to express HIV-1 Gag-Pol was measured using p24 ELISA (Lenti-X p24 Rapid Titer Kit, Takara Bio Cat. 631476) according to the manufacturer’s protocol. Culture supernatants were serially diluted ten-fold in a diluent buffer provided with the kit. Samples (100 µL) were added to the anti-p24 coated wells, followed by 20 µL of lysis buffer and a 30 min incubation. Then, 100 µL of HRP-conjugated anti-p24 antibody was added and incubated for another 30 min. Wells were washed six times with wash buffer, followed by addition of 100 µL tetramethylbenzidine (TMB) substrate. After a 30 min incubation protected from light, the reaction was stopped with 100 µL stop solution, and absorbance was read at 450 nm. p24 concentrations were interpolated from a standard curve (0–1600 pg/mL) constructed using the p24 control protein provided with the kit according to the manufacturer’s instructions.

### 2.9. Determination of Lentiviral Functional Titer

LV functional titers were determined by transducing HEK293FT cells with serial dilutions, followed by flow cytometric analysis of transduced cells. HEK293FT cells were seeded in 6-well plates (4 × 10^5^ cells per well) and allowed to attach. Serial dilutions of LV preparations were prepared in complete DMEM containing 8 µg/mL polybrene (Sigma Cat. H9268) and these dilutions were distributed in the wells to replace existing medium. Twelve hours later, the polybrene-containing medium was replaced with standard complete medium. Preliminary experiments were performed using a range of dilutions (1:10 to 1:10,000) to estimate the approximate titer. Subsequently, titrations were conducted using dilutions 1:10 to 1:1000 to ensure that 1–20% of cells were transduced in the last well [38]. At 72 h post-transduction, cells were harvested for analysis. For the LV/CAR-GFP vector, transduction efficiency was determined by quantification of GFP-positive cells. Cells were detached with trypsin-EDTA, washed with PBS, and resuspended in 1 mL of MACSQuant Running Buffer for cytometry (RB, Miltenyi Biotec Cat. 130-092-747). For the LV/CAR vector, non-permeabilized cells were stained by sequential incubation with biotinylated CD19 CAR Detection Reagent (Miltenyi Biotec Cat. 130-129-550) and anti-biotin-PE antibody (Miltenyi Biotec Cat. 130-113-291). Samples were acquired on a MACSQuant 10 flow cytometer (Miltenyi Biotec, Bergisch Gladbach, Germany). Above 50,000 events were acquired per sample. Data were analyzed using MACSQuantify Software (Miltenyi Biotec, Bergisch Gladbach, Germany) or FlowJo v10.8.1 (FlowJo, LLC, Ashland, OR, USA). Gates for positive cells were established using naïve HEK293FT cells as a negative control population, such that no more than 0.1% of events were detected within the positive gate during flow cytometry of naïve cells. In addition, MACSQuant Calibration Beads (Miltenyi Biotec Cat. 130-093-607) were used to monitor gate stability across experiments. The gating strategy is shown in Supplementary Material Figure S4. The functional titer (TU/mL) was calculated using the formula: TU/mL = (% positive cells/100) × (number of cells at transduction) × (dilution factor).

### 2.10. Lentiviral Vector Production in 5-Layer Stacks Using RCP or HEK293FT Cells

To directly compare the productivity of the RCP-based packaging platform with a conventional transient transfection system under identical conditions, parallel large-scale LV production runs were performed in 5-layer multilayer cell culture stacks (VWR, Cat. 734-3418; growth area 875 cm^2^, medium capacity 150 mL).

RCP-based production. HEK293FT-GP cells (replicon cell pool expressing HIV-1 Gag-Pol with the T26S mutation) were seeded into 5-layer stacks at a density of 1 × 10^5^ cells/cm^2^. Two hours before transfection, the culture medium was replaced with 150 mL of serum-free DMEM. Cells were co-transfected with a total of 450 μg of plasmid DNA using the calcium phosphate precipitation (CaPi) method. The plasmid mixture consisted of the transfer vector LV/CAR, the transactivator plasmid pTat/Rev, and the envelope plasmid pMD2.G at a molar ratio of 2:1:1 (corresponding to 200 μg transfer vector, 150 μg pTat/Rev, and 100 μg pMD2.G per stack). One hour after CaPi addition, fetal bovine serum (FBS) was added to a final concentration of 10%. Twelve hours later, the medium was replaced with 150 mL of fresh complete DMEM. The LV-containing supernatant was harvested 48 h post-transfection, clarified by centrifugation (1000× *g*, 10 min), and filtered through a 0.45 µm PES membrane (Pall Corporation Cat. 515-0157, Port Washington, NY, USA).

For conventional three-plasmid transient transfection, HEK293FT cells were seeded into five-layer stacks at a density of 1 × 10^5^ cells/cm^2^. Transfection, medium replacement, cell harvest, clarification, and filtration were performed following the same procedures described for RCP-based production. The second-generation LV packaging system was used consisting of the transfer vector LV/CAR, the packaging helper psPAX2 (Addgene Cat. 12260), and the envelope plasmid pMD2.G. The plasmids were transfected at a 2:1:1 molar ratio (LV/CAR/psPAX2/pMD2.G), with a total DNA amount of 450 μg per stack.

For both production systems, the clarified supernatants were processed as follows. LV particles were concentrated by tangential flow filtration (TFF) using a Tanfil 100 system (Rocker Scientific, Kaohsiung City, Taiwan) equipped with a 500 kDa membrane cartridge (Pall Life Sciences Cat. OA500C12, Port Washington, NY, USA). The supernatant was concentrated to approximately 7 mL, corresponding to the dead volume of the system. The concentrate was then centrifuged at 20,000× *g* for 2 h at 4 °C. The resulting pellets were left overnight in 0.5 mL of RPMI 1640 medium supplemented with 10% human serum albumin (HSA; Biopharma Plasma LLC, Bila Tserkva, Ukraine) and 0.0001% Pluronic F68 (Sigma Cat. P5556). The next day, the resuspended LV particles were pooled to a final volume of 500 µL and stored at −80 °C.

### 2.11. CAR-T Cell Manufacturing

To assess functional efficacy of a vector (LV/CAR) produced using YFV replicon-based packaging cell pools (HEK293FT-GP), we transduced primary human T-cells isolated from leukapheresis products as a practical target.

Source of starting material. Cryopreserved apheresis products were obtained from the Scientific and Production Center of Transfusiology (SPCT, Astana, Kazakhstan), a licensed clinical institution. Donors gave written informed consent at SPCT before collection. Only surplus frozen apheresis products unclaimed for clinical use were transferred to the National Center for Biotechnology (NCB); no material was collected specifically for this study. All products were provided to NCB as anonymized samples, without information allowing donor identification. This study protocol was approved by the NCB Institutional Ethics Committee.

Manufacturing process is described in detail in Keyer et al. [34]. A cryopreserved leukapheresis product was quickly thawed in a water bath (37 °C), diluted in warm RPMI medium (VWR Cat. 392-0429) supplemented with 50 U/mL Benzonase (Merck Cat. 70664-3), and incubated for 15 min at 37 °C. After centrifugation (300× *g*, 5 min) to remove cryoprotectants, the cell pellet was resuspended in 200 mL of RPMI supplemented with 3% human serum albumin (HSA). The resulting cell suspension (starting cell material) was aseptically transferred to a 300 mL transfer bag.

The subsequent CAR-T production steps were performed on a CliniMACS Prodigy cell processor (Miltenyi Biotec) according to the manufacturer’s instructions. The transfer bag (starting cell material) was sterilely welded to a TS520 Tubing Set (Miltenyi Biotec) installed on the CliniMACS Prodigy instrument. Other required consumables (all from Miltenyi Biotec) were also attached aseptically as instructed by the manufacturer.

The highly automated production process lasted 13 days.

On starting day (Day 0), the cell processor performed automated isolation of CD4^+^ and CD8^+^ T-cells using immunomagnetic separation according to a pre-programmed function of the CliniMACS Prodigy system. A total of 1 × 10^8^ purified CD4^+^ and CD8^+^ T-cells were seeded into the instrument’s incubation chamber. T-cell activation was initiated by adding the TransAct T Cell Activation Reagent (Miltenyi Biotec Cat. 130-128-758).

On the next day (Day 1), LV transduction was carried out using the pre-programmed “T-cell transduction (TCT 2.0)” procedure of the CliniMACS Prodigy instrument. For this purpose, the T cells were incubated with 2 × 10^8^ transducing units (TU) of LV/CAR. This vector was produced in this study using HEK293FT-GP replicon cell pools.

On Day 3, the culture medium was replaced with fresh medium, and the cells were then incubated until Day 12 according to a predefined automated expansion program (“activity matrix”). On Day 12, cells were washed and transferred into a final product bag. The final CAR-T cell product was adjusted to final concentrations of 10% HSA and 10% DMSO, then aliquoted into 1 mL portions and cryopreserved.

### 2.12. Functional Competence of Vector by Transduction of T Cells

The CAR-T cell product was analyzed by flow cytometry using the following antibody panel from Miltenyi Biotec: CD3-FITC (130-113-138), CD4-VioGreen (130-113-221), CD8-APC-Vio770 (130-113-155), CD14-APC (130-113-143), CD45-VioBlue (130-113-122), biotinylated CD19 CAR Detection Reagent (130-129-550), anti-biotin- phycoerythrin (PE)-conjugated antibody (130-113-291).

Staining for CAR detection was performed in two steps. First, 2 µL of biotinylated CD19 CAR Detection Reagent was added to 100 µL of cell suspension and incubated for 10 min at room temperature (RT). Then, 1 mL of MACSQuant Running Buffer (RB; Miltenyi Biotec Cat. 130-092-747) was added, and the cells were washed by centrifugation at 300× *g* for 10 min at RT. The supernatant was discarded, and the pellet was resuspended in 100 µL of RB. In the second step, a cocktail containing the panel antibodies listed above (except the CD19 CAR Detection Reagent) together with 2 µL of anti-biotin-PE antibody was added to the cells. After another 10 min incubation at RT, the cells were diluted with 1 mL of RB, centrifuged (300× *g* for 10 min) and finally resuspended in 1 mL of RB.

The percentage of CAR-positive (CAR^+^) cells, as well as the fractions of other hematopoietic cell types, were determined using a MACSQuant Analyzer 10 flow cytometer. Flow cytometry data were analyzed with MACSQuantify Software v.2.13 (including the Express Modes module; Miltenyi Biotec, Bergisch Gladbach, Germany) and FlowJo v10.8.1 (FlowJo, LLC, Ashland, OR, USA).

### 2.13. Assessment of Gag-Pol Expression During RCP Passages

To evaluate whether the replicon cell pool HEK293FT-GP maintains sufficient Gag-Pol expression for practical scale-up, two experiments were performed, each in three biological replicates.

Experiment 1 (continuous puromycin selection). HEK293FT-GP cells were cultured in complete DMEM supplemented with 10 µg/mL puromycin for ten consecutive passages (P1 to P10). The initial passage (designated P0) was derived from cryopreserved HEK293FT-GP cells restored in the presence of puromycin. At each passage, cells were seeded at 5 × 10^5^ cells per 10 cm dish and incubated for 48 h. Culture supernatants were collected, clarified by centrifugation (1000× *g*, 10 min), and stored at −80 °C. The concentration of HIV-1 p24 capsid protein (a surrogate for Gag-Pol-derived VLP production) was quantified using the Lenti-X p24 Rapid Titer Kit (Takara Bio Cat. 631476) according to the manufacturer’s instructions. p24 levels (ng/mL) were interpolated from a standard curve (0–1600 pg/mL). The goal of this experiment was to determine whether the VLP yields (indicator of Gag-Pol expression) remained statistically unchanged throughout the passages needed to expand the culture to generate sufficient cells for seeding one to three multilayer culture stacks.

Experiment 2 (withdrawal and re-introduction of selection). HEK293FT-GP cells from cryopreservation were reconstituted in the presence of puromycin (passage P0). These cells were then propagated without puromycin for five passages (P1–P5). Puromycin (10 µg/mL) was then re-introduced at passage P6 and maintained through passage P12. Culture supernatants were collected at P1–P5 (without selection) and at P8–P12 (after re-introduction of selection) and analyzed for p24 by ELISA. The goal of this experiment was to determine whether VLP production levels declined in the absence of selection, indicating loss of or changes in the replicon; and to assess whether the productive phenotype could be restored following a phase of selection withdrawal by re-introducing puromycin.

All experiments were performed with three independent replicate cultures per condition.

### 2.14. Sequencing of the Gag-Pol Insert in the Replicon After Sequential Passages

Total RNA was extracted from replicon-bearing cells at passage P10 (obtained from Experiment 1, as described in Section 2.13) using TRIzol Reagent (Thermo Scientific Cat. 15596026, Waltham, MA, USA) according to the manufacturer’s instructions. Half of the total RNA from a P100 dish was reverse-transcribed into cDNA using SuperScript II Reverse Transcriptase (Thermo Scientific Cat. 18064014) with random hexamer primers. PCR was performed using Phusion High-Fidelity DNA Polymerase (Thermo Scientific, Cat. F530L) and the primers targeting the Gag-Pol insert in the replicon (see Supplementary Material Table S1). PCR products were purified with AMPure XP magnetic beads (Beckman Coulter Cat. A63881, Brea, CA, USA) following the manufacturer’s protocol. The purified products were subjected to bidirectional Sanger sequencing. The resulting contigs were compared with the parental MIC to detect mutations.

### 2.15. Replication-Competent Lentivirus (RCL) Detection Assay

RCL in vector preparations was assessed using C8166 cells as an indicator cell line following published protocols [39,40,41]. The assay was initiated by transducing 2 × 10^5^ C8166 cells in a T25 flask with an aliquot of the final concentrated LV/CAR product corresponding to 5% of the final volume, which amounted to 2.18–3.28 × 10^7^ transducing units (TU) per test. After overnight incubation with the LV inoculum, the medium was removed by centrifugation (300× *g*, 5 min), and the cells were resuspended in fresh complete medium.

The transduced cells were serially passaged every 2–3 days for a total of 21 days, resulting in 10 culture passages (P1–P10, RCL amplification phase). After each passage, cells were centrifuged (300× *g*, 5 min), resuspended in fresh complete medium, counted, and seeded at a density of approximately 1 × 10^6^ cells into a new T25 flask containing 10 mL of fresh complete RPMI-1640 medium. The supernatant of conditioned medium was collected and tested by p24 ELISA (Lenti-X p24 Rapid Titer Kit, Takara Bio Cat. 631476).

After the 21-day amplification phase, cell-free culture supernatant was produced by centrifugation (300× *g*, 5 min) and filtration through a 0.45 µm filter. This clarified supernatant (1 mL aliquot) was then used to inoculate fresh naïve C8166 cells (2 × 10^5^ cells per T25 flask) for an additional 7-day indicator phase [39,40,41]. At the end of the indicator phase, the culture supernatant was analyzed by p24 ELISA.

A vector batch was considered free of detectable RCL if p24 levels during the amplification phase showed a progressive decline, and p24 concentration in the indicator phase remained below the limit of detection (LOD). The LOD was defined as the p24 concentration (pg/mL) corresponding to the mean OD_450_ of conditioned medium from naïve C8166 cells plus three standard deviations, calculated using the standard curve of the p24 ELISA (see Supplementary Material Table S2 for representative calculations).

### 2.16. Statistical Analysis

Three biological replicates were done unless otherwise stated. Data are presented as mean ± standard deviation (SD). MTT assay results were analyzed using an unpaired two-tailed Student’s *t*-test. p24 production in serial passages was compared using one-way repeated measures ANOVA with the Geisser–Greenhouse correction. Statistical analyses were carried out using GraphPad Prism version 9.3.1 (GraphPad Inc., San Diego, CA, USA). Significance levels are denoted as: *ns* (*p* > 0.05), * (*p* ≤ 0.05), ** (*p* ≤ 0.01), *** (*p* ≤ 0.001).

## 3. Results

### 3.1. Engineering YFV Replicons for Transient Gag-Pol Expression

**Figure 1:**
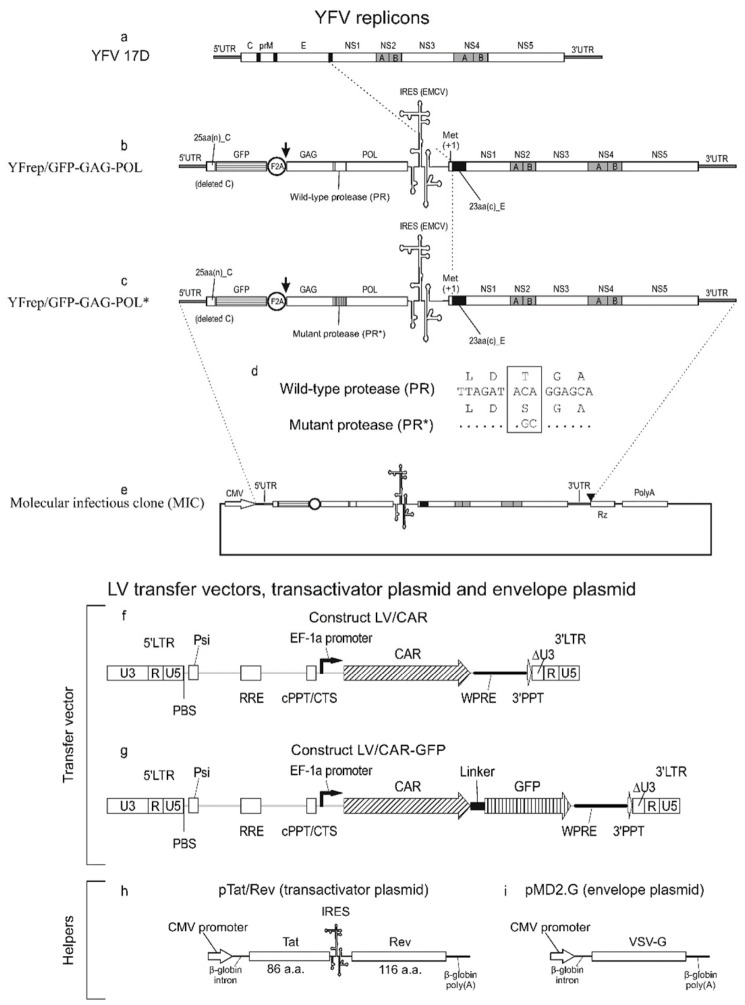
Schematic representation of yellow fever virus (YFV) replicons and different constructs used in this study. (**a**) Genome organization of the yellow fever virus (YFV). The genome includes 5′ and 3′ untranslated regions (UTRs) and a long open reading frame encoding structural (C-prM-E) and non-structural (NS1-NS5) proteins. (**b**,**c**) YFV-derived replicons expressing GFP and HIV-1 Gag-Pol. The structural genes are replaced by a bicistronic gene cassette. The essential cyclization signal within the N-terminal 25 amino acids of the capsid protein (25aa(n)_C) is retained. Black downward arrows indicate the positions of F2A self-cleaving peptide cleavage sites. (**b**) Schematic of the replicon YFrep/GFP-GAG-POL. Replicons are fragments of the viral genome in which structural protein genes are removed, replaced by heterologous genes. The capsid gene (C) is removed except for a fragment encoding the N-terminal 25 amino acids (25aa(n)_C) that contains the essential cyclization signal. The gene cassette includes the green fluorescent protein (GFP) gene, followed by the foot-and-mouth disease virus 2A (F2A) self-cleaving peptide and the full-length HIV-1 Gag-Pol genes. Gag encodes HIV-1 capsid proteins and is translated as the major translation product. The Gag-Pol fusion is generated via a -1 ribosomal frameshift at the GAG/POL junction. The Pol product is processed into individual HIV-1 enzymes. In this replicon, the Pol product includes the wild-type HIV-1 protease (PR). Downstream of the Pol stop codon, an encephalomyocarditis virus internal ribosome entry site (EMCV IRES) and an engineered Met+1 start codon are inserted. The IRES controls translation of the YFV non-structural proteins NS1-NS5, starting from the C-terminal 23 amino acids of the E protein (23aa(c)_E), which serve as a signal peptide required for the translocation and processing of NS1. (**c**) Replicon YFrep/GFP-GAG-POL* is identical to (**b**) except that, in the GFP-F2A-Gag-Pol-IRES gene cassette, the Pol gene encodes a mutant HIV-1 protease (PR*) carrying an attenuating T26S substitution. (**d**) Comparison of wild-type HIV-1 protease (PR) and the T26S mutant (PR*). The position of the T26S substitution is indicated by the altered amino acid residue (T→S) and the corresponding nucleotide change (ACA→AGC). (**e**) Schematic representation of the DNA-launched molecular infectious clone (MIC). The replicon sequence is cloned into a bacterial plasmid (shown as a rectangle) under the control of the human cytomegalovirus (CMV) immediate-early promoter. Following the viral 3′ terminus, the antigenomic ribozyme of hepatitis delta virus (Rz) and the polyadenylation signal of the human growth hormone gene (PolyA) ensure proper 3′ end processing. The black downward arrowhead indicates the position of ribozyme cleavage, which generates the authentic 3′ end of the replicon RNA. (**f**) Map of the transfer vector for expression of chimeric antigen receptor (CAR). Shown are the 5′ and 3′ long terminal repeats (LTRs; 3′LTR with a deletion in U3, ΔU3), the packaging signal (Psi), Rev response element (RRE), central polypurine tract (cPPT), and the EF-1α promoter driving expression of the transgene. WPRE: woodchuck hepatitis virus posttranscriptional regulatory element. (**g**) Map of the LV transfer vector for expression of CAR and GFP. (**h**) Map of the transactivator plasmid pTat/Rev. The CMV promoter drives expression of a bicistronic transcript encoding HIV-1 Tat and Rev, linked by an EMCV IRES. The β-globin intron and poly(A) signal are also shown. (**i**) Map of the envelope plasmid pMD2.G. The CMV promoter drives expression of the VSV-G glycoprotein. β-globin intron and poly(A) signal are indicated. Abbreviations: PR, wild-type HIV-1 protease; PR*, mutant HIV-1 protease (T26S); F2A/T2A, self-cleaving peptides; IRES, internal ribosome entry site; Pac, puromycin acetyltransferase; CMV, cytomegalovirus promoter; Rz, ribozyme; PolyA, polyadenylation signal; LTR, long terminal repeat; RRE, Rev response element; cPPT, central polypurine tract; WPRE, woodchuck hepatitis virus posttranscriptional regulatory element; VSV-G, vesicular stomatitis virus G glycoprotein.

For the initial proof of concept, replicons derived from the yellow fever virus (YFV) were engineered. The organization of the YFV genome is depicted in Figure 1a. A replicon is a portion of the viral genome capable of autonomous replication in the cytoplasm. Accordingly, in all our replicons, the structural genes are replaced with a bicistronic cassette, while the 5′ and 3′ untranslated regions (UTRs), the essential cyclization signal within the N-terminal 25 amino acids of the YFV capsid protein (25aa(n)_C), and the replicase genes (NS1–NS5) are retained.

We first engineered two GFP-expressing replicons for transient expression and cytotoxicity studies: YFrep/GFP-GAG-POL and YFrep/GFP-GAG-POL* (Figure 1b,c). In these constructs, the YFV sequences downstream of the cyclization signal are fused to GFP, followed by an F2A self-cleaving peptide and the full-length HIV-1 Gag-Pol polyprotein. Downstream of the Pol stop codon, an IRES element drives translation of the YFV non-structural proteins NS1-NS5, providing the RNA polymerase required for autonomous replication. The replicon YFrep/GFP-GAG-POL carries the wild-type HIV-1 protease (PR), while YFrep/GFP-GAG-POL* contains the attenuating T26S substitution in the protease (PR); the alignment fragment in Figure 1d highlights the differences between PR and PR*.

The replicons were cloned into DNA-launched molecular infectious clones (MICs) (Figure 1e) containing eukaryotic transcription control elements (CMV promoter, ribozyme, polyA signal), which ensure proper 5′- and 3′-termini formation upon transcription and initiate replicon replication upon MIC DNA transfection.

### 3.2. The T26S Protease Mutation Reduces Replicon Cytotoxicity and Affects Gag Processing

**Figure 2:**
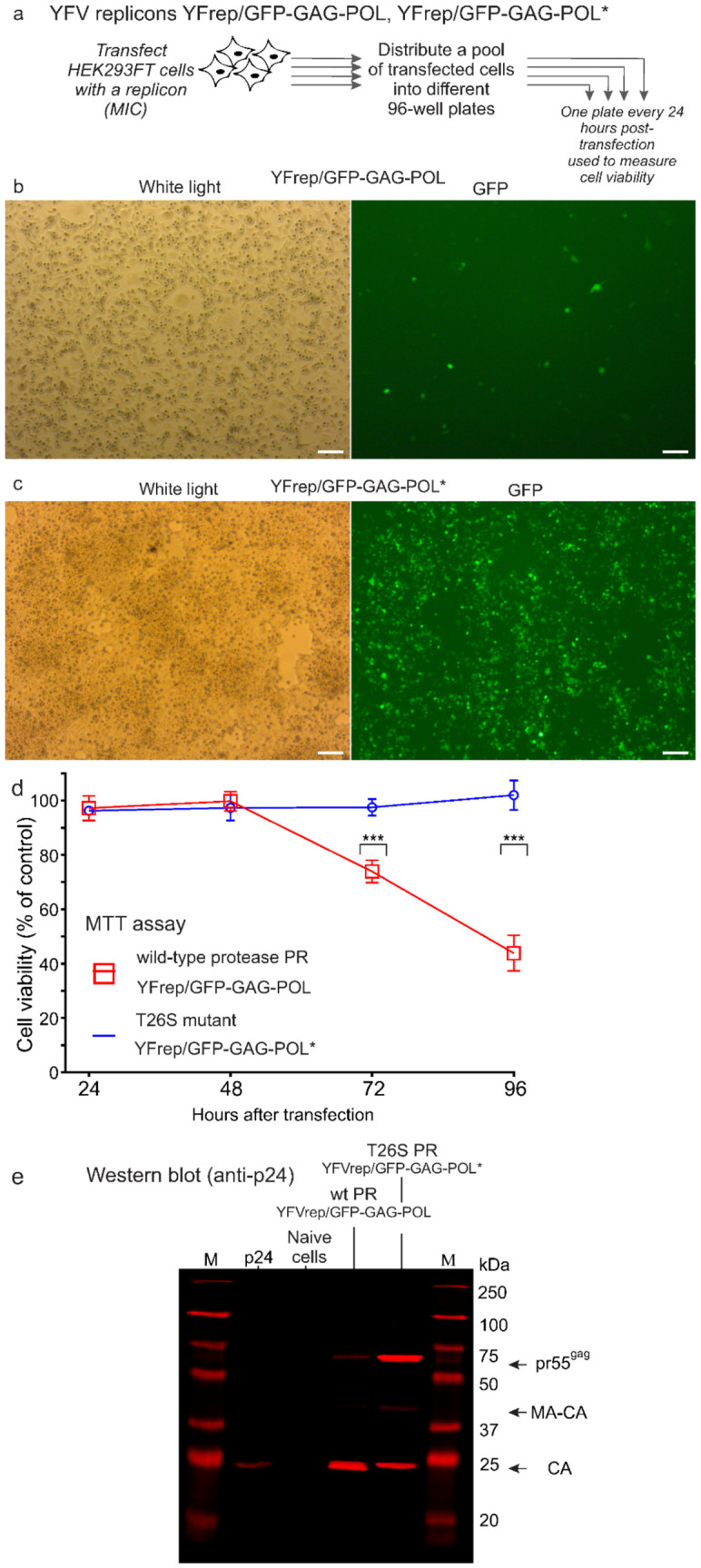
Cytotoxicity and Gag processing in HEK293FT cells upon transfection with Gag-Pol-expressing YFV replicons. (**a**) Schematic of the experimental workflow. HEK293FT cells were transfected with YFrep/GFP-GAG-POL (wild-type protease PR) or YFrep/GFP-GAG-POL* (T26S mutant protease PR*). Cytotoxicity and Gag processing were assessed at the indicated time points. (**b**) Cells transfected with pYFrep/GFP-GAG-POL, 72 h after transfection. Phase-contrast (**left**) and GFP fluorescence (**right**) images show cytopathic effect (CPE), manifesting in cell rounding, detachment, and weak GFP signal. Magnification: 10× objective; scale bar: 100 µm. (**c**) Cells transfected with pYFrep/GFP-GAG-POL*, 72 h after transfection. No advanced CPE was observed. Cells resumed proliferation and reached confluence by 96 h. Magnification: 10× objective; scale bar: 100 µm. (**d**) Cell viability was assessed by MTT assay. Data are presented as percentage of control, where mock-transfected cells were set to 100% at each time point. Viability of cells transfected with the wild-type protease replicon (pYFrep/GFP-GAG-POL) began to decline at 72 h post-transfection. In contrast, cells expressing the T26S mutant protease (pYFrep/GFP-GAG-POL*) maintained viability comparable to mock-transfected controls throughout the 96 h observation period. Data points represent the mean of three independent biological replicates (each with four technical replicates, total 12 wells per condition). Error bars are SD. Statistically significant differences between the wild-type and mutant replicons at 72 h and 96 h are indicated: *** *p* ≤ 0.001 (Student’s *t*-test). (**e**) Western blot analysis of Gag processing in HEK293FT cells bearing the replicons YFrep/GFP-GAG-POL and YFrep/GFP-GAG-POL*. Cell lysates were harvested 48 h post-transfection and analyzed using an anti-p24 primary antibody and an IRDye 680RD-labeled secondary antibody. The membrane was scanned on a LI-COR Odyssey system in the 680 nm channel. Lanes (left to right): recombinant p24 (positive control), naïve HEK293FT cells (negative control), cells transfected with YFrep/GFP-GAG-POL (wild-type PR), cells transfected with YFrep/GFP-GAG-POL* (T26S mutant PR). M, Bio-Rad Precision Plus Protein Standard (Cat. #1610373); molecular masses (kDa) are indicated on the right. The wild-type PR replicon directed efficient Gag cleavage, yielding abundant mature p24 and minimal residual pr55^gag^ precursor. Conversely, the T26S mutant PR replicon exhibited accumulation of the pr55^gag^ precursor and persistence of intermediate cleavage products (MA-CA), with a corresponding reduction in mature p24. This experiment was repeated with qualitatively consistent results. The full membrane image is shown in Supplementary Material Figure S5.

To evaluate whether the engineered replicons could establish replication and express the heterologous proteins from inserted genes, HEK293FT cells were transiently transfected with the MICs pYFrep/GFP-GAG-POL (wild-type PR) or pYFrep/GFP-GAG-POL* (T26S mutant PR) and monitored for GFP expression and cytopathic effect (CPE). The experimental scheme is shown in Figure 2a. Following MIC transfection, the replicon RNA was expected to be transcribed in the nucleus, exported to the cytoplasm, and initiate autonomous replicon replication. GFP fluorescence was detected in both cultures at 48–72 h post-transfection (Figure 2b,c), confirming RNA replication and transgene expression.

Cells bearing the wild-type protease replicon exhibited increasing CPE starting at 72 h post-transfection, characterized by an increasing fraction of visibly dead cells which rounded and detached (Figure 2b, left panel). In this culture, fewer cells expressed GFP compared to the culture bearing the T26S mutant replicon (left panel in Figure 2c). Cells expressing the mutant PR showed no obvious CPE, resumed normal proliferation, and reached confluence by 96 h. Moreover, a higher fraction of cells in the mutant replicon culture demonstrated GFP fluorescence (compare right panels in Figure 2b,c).

MTT assay showed that viability of a culture carrying the wild-type PR replicon began to decline at 72 h post-transfection (Figure 2d). In contrast, viability of cells carrying the mutant PR* replicon remained comparable to mock-transfected controls throughout the 96 h observation period. These results demonstrate that the T26S mutation reduces replicon-induced CPE to levels undetectable by the MTT assay.

Processing of the HIV-1 Gag polyprotein expressed in cells infected with the wild-type PR replicon and the mutant PR* replicon was analyzed by Western blot using an anti-p24 antibody (Figure 2e). In cells expressing wild-type PR, efficient processing of the Gag precursor was observed, as evidenced by the predominance of the mature p24 capsid band (Figure 2e). In contrast, cells expressing the T26S mutant PR* showed impaired Gag processing, characterized by accumulation of the unprocessed pr55^gag^ precursor and persistence of intermediate cleavage products, with a corresponding reduction in the mature p24 band. These qualitative differences were confirmed in independent blotting experiments with consistent results and indicate that the T26S mutation attenuates, but does not eliminate, protease activity, resulting in reduced Gag processing. These findings are consistent with the previously published observations of Konvalinka et al. [42].

### 3.3. Construction of Selectable Replicons and Establishment of Replicon Cell Pools

**Figure 3:**
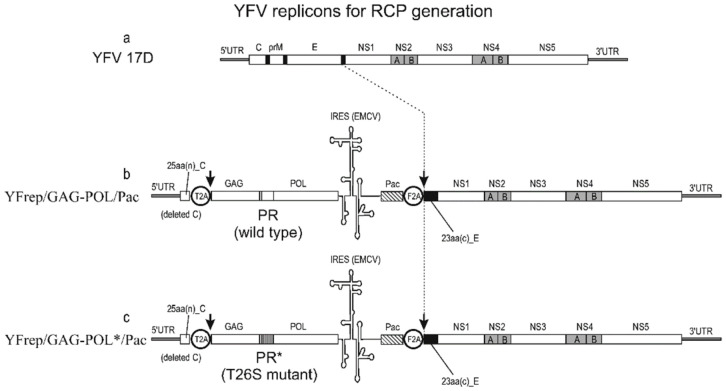
Construction of replicons for the expression of HIV-1 Gag-Pol and puromycin acetyltransferase. (**a**) Genome of YFV showing structural (C-prM-E) and non-structural (NS1-5) proteins. (**b**) Replicon YFrep/GAG-POL/Pac: The capsid protein (C) is removed, retaining only a cyclization signal. The fragment of the capsid protein (25aa(n)_C) is fused via the T2A self-cleaving peptide to the HIV-1 Gag-Pol polyprotein. The Gag protein is the in-frame translation product, while the Pol part in the Gag-Pol polyprotein is generated via a ribosomal frameshift. Downstream of the native POL stop codon, an encephalomyocarditis virus internal ribosome entry site (EMCV IRES) is inserted. This IRES directs translation of puromycin acetyltransferase (Pac), through an F2A self-cleaving peptide, fused to the C-terminal 23 amino acids of the YFV E protein (23aa(c)_E), which serves as a signal sequence for NS1 translocation. The F2A-mediated cleavage releases Pac-2A as a separate protein while allowing translation to continue into the NS1-NS5 region. (**c**) Replicon YFrep/GAG-POL*/Pac: This replicon is similar to (**b**) but contains the mutant protease (T26S). Downward black arrows indicate cleavages mediated by self-cleaving peptides. UTR, untranslated regions; T2A, *Thosea asigna* virus 2A self-cleaving peptide; F2A, foot-and-mouth disease virus 2A self-cleaving peptide; Pac, puromycin acetyltransferase.

To confer antibiotic selection properties, new versions of the replicons were engineered to carry a gene encoding puromycin acetyltransferase (Pac) (Figure 3). In these replicons, YFrep/GAG-POL/Pac and YFrep/GAG-POL*/Pac, the first cistron encodes the N-terminal capsid fragment fused via a T2A peptide to Gag-Pol, either in its wild-type form or as the T26S mutant. The second cistron, placed under the control of an internal ribosome entry site (IRES), encodes Pac followed by an F2A peptide and the YFV NS1–NS5 proteins (Figure 3b,c). Following F2A-mediated cleavage, Pac is released to confer puromycin resistance, while translation continues into the NS1–NS5 region.

**Figure 4:**
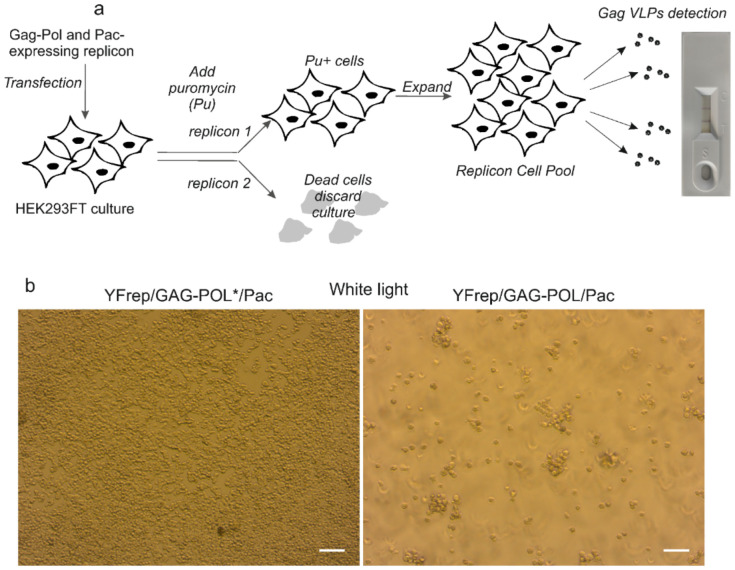
Establishment of a replicon cell pool (RCP). (**a**) Schematic of the experiment: HEK293FT cells were transfected with YFV replicons that express HIV-1 Gag-Pol and puromycin acetyltransferase (Pac). Two days post-transfection, puromycin (10 µg/mL) was added to the culture medium. Cells harboring a functional replicon that provides puromycin resistance survive selection and can be expanded to form a polyclonal replicon cell pool (RCP). Production of Gag virus-like particles (VLPs) from such cells confirms Gag-Pol expression, as demonstrated by the positive Lenti-X GoStix Plus strip shown. (**b**) Representative phase-contrast micrographs of HEK293FT cultures after 24 h of puromycin selection. (**Left**): cells bearing the replicon YFrep/GAG-POL*/Pac, which encodes the T26S mutant HIV-1 protease (PR*); most cells survived. Subsequently, cells resumed proliferation. (**Right**): cells transfected with the wild-type protease replicon YFrep/GAG-POL/Pac; the majority of cells died and detached, precluding establishment of a replicon cell pool. Magnification: 10× objective; scale bar: 100 µm.

HEK293FT cells were transfected with the MIC pYFrep/GAG-POL*/Pac (T26S mutant protease) or its wild-type counterpart. Puromycin (10 µg/mL) was added 48 hours post-transfection (Figure 4a). Cells expressing the mutant replicon survived selection, resumed growth, and were successfully expanded and cryopreserved, yielding a polyclonal replicon cell pool designated HEK293FT-GP (Figure 4b, left photograph). In contrast, the majority of cells transfected with the wild-type protease replicon died and detached within 24–48 h after puromycin addition (Figure 4b, right photograph), and these cultures were discarded.

To confirm functional Gag-Pol expression, we assessed the production of virus-like particles (VLPs) into the culture medium of replicon cell pool. Immunochromatographic strips, which detect virion-associated p24 protein, revealed p24 in culture supernatants of HEK293FT-GP cells (Figure 4).

### 3.4. Gag-Pol Expression in RCP Cells During Serial Passaging with and Without Selection

**Figure 5:**
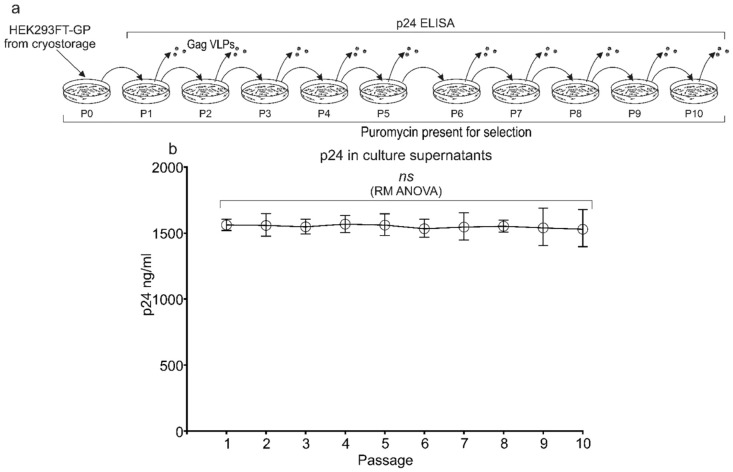
p24 production in culture supernatants of the replicon cell pool HEK293FT-GP during consecutive passages under puromycin selection. (**a**) Experimental scheme: HEK293FT-GP cells were cultured continuously in the presence of puromycin (10 µg/mL). On day 2 of each passage, cells were reseeded into fresh culture dishes. Culture supernatants were collected before reseeding. Three independent replicate lines were maintained under identical conditions. p24 levels were quantified by ELISA. (**b**) p24 concentrations (ng/mL) in culture supernatants from passages P1–P10 (*n* = 3 per passage). The graph shows mean values ± SD. A one-way repeated measures ANOVA (RM ANOVA) showed no significant effect of passage number on p24 concentration (*ns*, *p* > 0.05).

To examine whether the established RCP cells maintain Gag-Pol expression over passages under continuous selection, HEK293FT-GP cells were cultured in the presence of puromycin (10 µg/mL) for passages P1–P10. At each passage, culture supernatants were collected and p24 concentrations were quantified by ELISA (Figure 5a,b). Three independent RCP cell lines were passaged to enable statistical analysis. p24 concentrations ranged from 600 to 700 ng/mL across different samples, but no dependence on passage number was observed (Figure 5b). A one-way repeated measures ANOVA (RM ANOVA) confirmed no significant effect of passage number (*ns*, *p* > 0.05).

**Figure 6:**
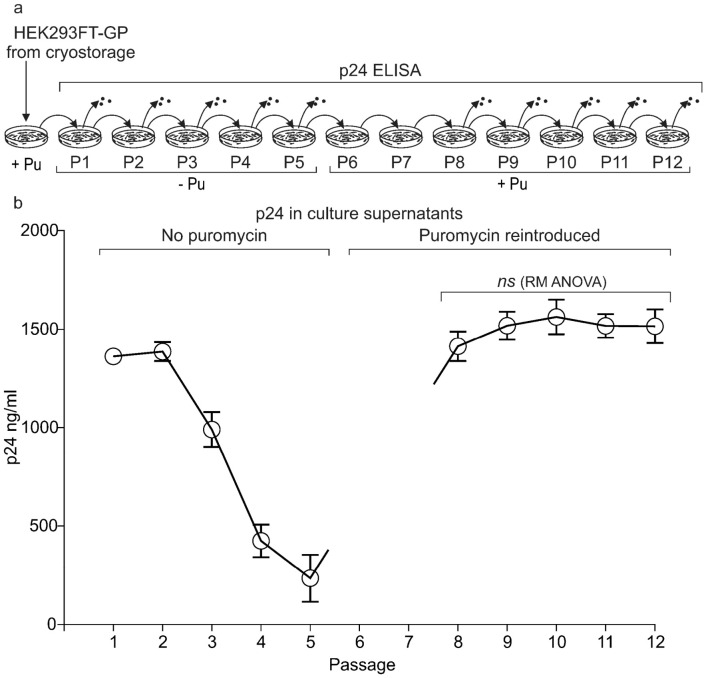
p24 production in culture supernatants by RCP cells grown without selection and after reintroduction of selection. (**a**) RCP cells were established under puromycin (Pu) selection. Pu was withdrawn for five passages (P1–P5) and reintroduced from passages P6–P12. Supernatants were collected at passages P1–P5 and P8–P12. Cells were reseeded on day 2 after seeding, and supernatants were harvested 48 h prior to reseeding. Three independent replicate lines were analyzed, and p24 levels were quantified. (**b**) p24 concentrations (ng/mL) in culture supernatants. Data are shown as means ± SD. The gap in the curve represents two passages (P6–P7) during which p24 production was not measured. A one-way repeated measures ANOVA (RM ANOVA) performed on passages P8–P12 (after antibiotic selection was reintroduced) showed no significant effect of passage number on p24 concentration (*ns*, *p* > 0.05).

To examine whether the RCP requires continuous antibiotic selection to maintain replicon-mediated Gag-Pol expression, we transferred the established puromycin-resistant HEK293FT-GP cells into medium without the antibiotic for five passages (P1–P5, Figure 6a). Puromycin was then reintroduced at passage P6 and maintained through P12. Culture supernatants were collected at passages P1–P5 (without selection) and at passages P8–P12 (after re-exposure to puromycin), and p24 concentrations were quantified by ELISA.

As shown in Figure 6b, p24 production gradually declined during the five passages without puromycin selection, indicating that either a fraction of cells lost the replicon, or the replicon in a subset of cells lost the ability to produce Gag-Pol. However, after puromycin was reintroduced (P8–P12), p24 levels returned to values previously measured for cells under selection (presented in Figure 5). For passages P8–P12, a repeated measures ANOVA revealed no significant effect of passage number on p24 concentration (*p* > 0.05).

These results demonstrate that maintenance of Gag-Pol expression depends on antibiotic selection. at least 10^8^–10^9^ cells, sufficient for seeding multiple multilayer culture stacks for LV production. This illustrates the productive capacity of the RCP approach and suggests that, with further optimization, improved RCPs could occupy an intermediate niche between conventional transient transfection and stable PCL approaches.

The presented results indicate that sequential passaging of cryopreserved HEK293FT-GP cells under selection in expanding cultures can generate 10^8^–10^9^ cells within a limited number of passages (e.g., ten), sufficient to seed multiple multilayer culture stacks for LV production. Collectively, these findings demonstrate the productive capacity of the RCP approach and suggest that, with further optimization, RCP systems may occupy an intermediate niche between conventional transient transfection and stable PCL-based production strategies.

### 3.5. Sequencing Reveals No Mutations in the Gag-Pol Insert

This result indicates that the HIV-1-specific genes in the YFV replicon did not accumulate mutations over ten passages, and this stability may be attributed to properties of the YFV replicon itself.

To assess whether prolonged passaging of the RCP under selective pressure leads to accumulation of mutations in the HIV-1-derived sequences, we performed RT-PCR and Sanger sequencing of the entire Gag-Pol insert after ten consecutive passages in the presence of puromycin (Experiment 1 described in Section 3.4). No nucleotide substitutions were detected in the sequenced region compared to the parental MIC, and the attenuating T26S mutation remained present. This result demonstrates that the YFV replicon maintained the genetic integrity of the heterologous Gag-Pol insert over at least ten passages, corresponding to approximately 25–30 cell doublings.

### 3.6. Production of Lentiviral Vectors Using Replicon Cell Pools (RCPs) or Conventional Three-Plasmid Transfection of HEK293FT Cells

**Figure 7:**
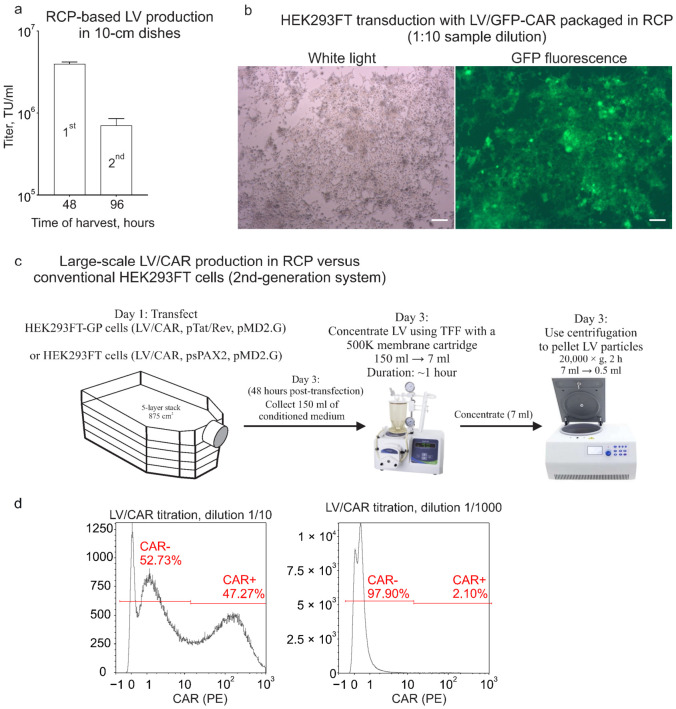
Production and titration of LV using replicon cell pools (RCPs) or conventional three-plasmid transfection of HEK293FT cells. (**a**) LV/CAR-GFP production in 10 cm dishes. Supernatant was harvested at 48 h post-transfection, replaced with fresh medium, and collected again at 96 h. Bar graph shows functional titers (TU/mL) from three independent experiments (mean ± SD). (**b**) Representative images of HEK293FT cells transduced with LV/CAR-GFP during titration experiment. Cells in the first well of a 6-well plate (1:10 dilution of culture supernatant) were photographed 48 h post-transduction. Left: phase contrast; right: GFP fluorescence. Magnification: 10× objective; scale bar: 100 µm. (**c**) Schematic workflow of large-scale LV/CAR production in 5-layer stacks using RCP cells, or conventional HEK293FT cells. (**d**) Flow cytometry histograms for titration of LV/CAR vector. HEK293FT cells were transduced to express CAR and stained with PE-conjugated anti-CAR antibodies. Numbers indicate the percentage of CAR-positive cells.

To evaluate the productivity of the RCP-based packaging system, we first packaged the GFP-tagged vector LV/CAR-GFP in P100 dishes using HEK293FT-GP cells (Figure 7a). RCP cells were transfected with the transfer vector LV/CAR-GFP and two packaging plasmids as described in Materials and Methods Section 2.10. The first harvest of vector-containing supernatant was collected at 48 h post-transfection. The cultures were then replenished with fresh medium, and a second harvest was collected at 96 h. The first harvest yielded mean titers of 3.95 × 10^6^ TU/mL (range 3.78–4.22 × 10^6^ TU/mL, n = 3), whereas the second harvest gave substantially lower titers representing approximately 18% of the first-harvest yield (Figure 7a). Accordingly, only a single harvest was used in subsequent experiments. The ability of the RCP-produced LV/CAR-GFP vector to transduce target cells was demonstrated in the titration experiment by GFP fluorescence. Representative images showing clear GFP expression at 48 h post-transduction are presented in Figure 7b.

We next scaled up production to 5-layer culture stacks (growth area 875 cm^2^) and compared the RCP-based platform with a conventional second-generation transient transfection system (Figure 7c). For RCP-based production, HEK293FT-GP cells were transfected with LV/CAR, pTat/Rev, and pMD2.G plasmids. In the conventional system, naïve HEK293FT cells were transfected with LV/CAR, psPAX2, and pMD2.G. All other production parameters were kept identical to enable direct comparison. Functional titers (TU/mL) were determined at each stage of the production process. Representative flow cytometry histograms from titration of the RCP-packaged LV/CAR vector are shown in Figure 7d.

The functional titers obtained with the two packaging systems at different process stages are presented in Table 1. In two independent experiments, the unconcentrated supernatants from the RCP system yielded functional titers (4.20 × 10^6^ and 6.06 × 10^6^ TU/mL) that were within the same order of magnitude as those obtained with the conventional system (8.01 × 10^6^ and 8.23 × 10^6^ TU/mL). Due to the limited number of production runs, statistical comparison was not performed, and these data should be interpreted as preliminary. Additional experiments will be required to definitively compare the RCP platform with conventional transient transfection; nevertheless, given the substantial potential for further improvement of the RCP system, future comparisons would be more appropriately conducted using a further optimized version of the platform. After all concentration steps, the final LV preparations from the conventional system showed slightly higher titers (Table 1), which may indicate greater particle stability in that system. Nevertheless, additional experiments are required to confirm this observation.

To assess the specific infectivity of the final LV/CAR preparations, virion-associated p24 was quantified by ELISA, and functional titers were used to calculate TU/pg p24. The TU/pg p24 values ranged from 379 to 472 for RCP-produced vectors and from 710 to 907 for the conventional system (Table 2). These results indicate higher TU/pg p24 values for the conventional packaging system compared with the RCP-based platform under the conditions tested. Statistical analysis was not performed due to the limited number of experiments; therefore, additional studies are required to confirm this observation.

### 3.7. Production of CAR-T Cells Using the Vector Produced with RCP

**Figure 8:**
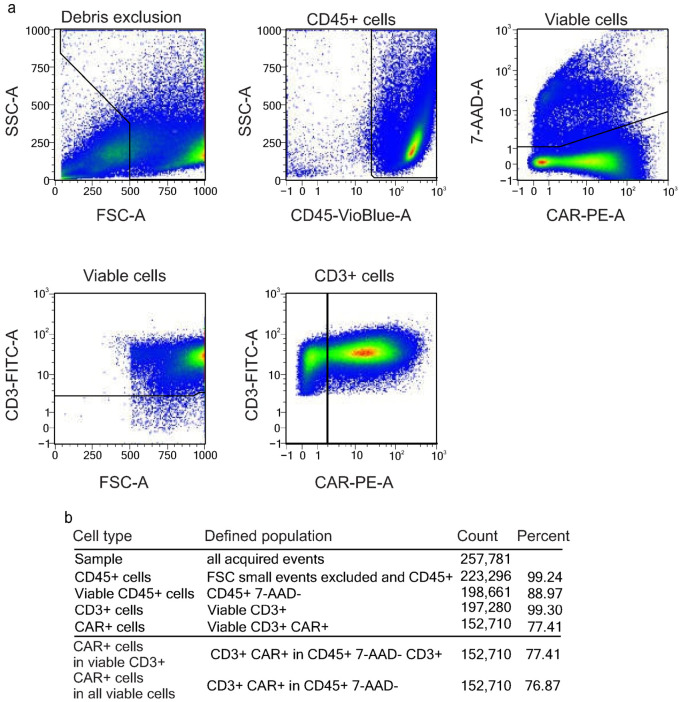
Characterization of a CAR-T cell product generated using LV/CAR vector produced from RCP cells. (**a**) Representative flow cytometry plots showing the gating strategy used for counting CAR^+^ cells (debris exclusion, CD45^+^ leukocytes, viable cells, CD3^+^ T cells, and CAR^+^ subset within CD3^+^ cells). (**b**) Summary of cell product composition after T-cell selection and transduction, including cell subsets, markers, event counts, and relative frequencies.

To assess whether an LV packaged using YFV replicon-based HEK293FT-GP cells is functionally competent for CAR-T cell manufacturing, automated production was performed using the CliniMACS Prodigy instrument, as described in Materials and Methods (Section 2.11). Primary human CD4^+^ and CD8^+^ T lymphocytes were isolated from a donor leukapheresis product by immunomagnetic separation, activated for proliferation, and transduced with the LV/CAR vector at a multiplicity of infection (MOI) of 2. Cell product characteristics were analyzed on day 5 post-transduction by flow cytometry, including determination of CAR^+^ cell populations (Figure 8). The complete dataset is provided in the Supplementary Material Figure S6. The proportion of CAR^+^ cells among viable CD3^+^ cells was 77.41%. These results indicate that the RCP-produced LV/CAR vector efficiently transduces primary human T cells and supports generation of CAR-T cell products.

### 3.8. Assessment of Replication-Competent Lentivirus (RCL)

**Figure 9:**
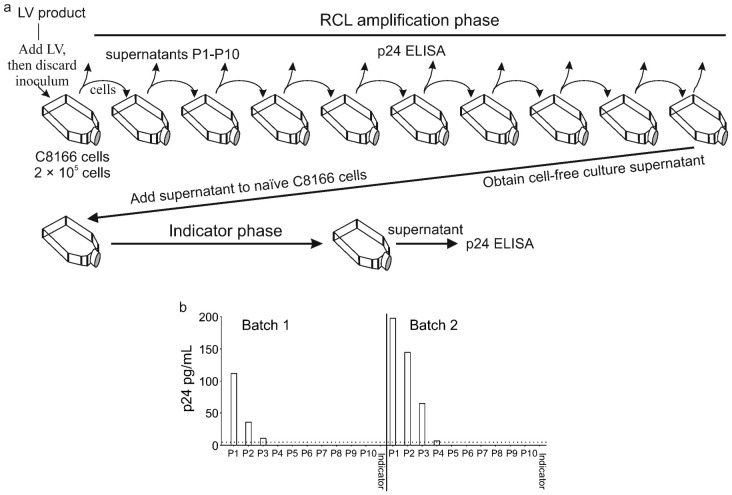
Detection of replication-competent lentivirus. (**a**) Schematic workflow: An aliquot of the final LV/CAR product was used to transduce C8166 cells. Cells were passaged every two days for 21 days (10 passages). Cell-free supernatant from this amplification phase was then used to inoculate naïve C8166 cells for an additional 7-day indicator phase. Culture supernatants were quantified for HIV-1 p24 by ELISA. (**b**) p24 concentrations (pg/mL) at passages P1–P10 (amplification phase) and after the indicator phase. Values below the LOD (dashed line) are not shown. The LOD was defined as p24 concentration (pg/mL) corresponding to the mean OD_450_ of conditioned medium from naïve C8166 cells plus three standard deviations (Supplementary Table S2).

To evaluate the presence of detectable RCL in LV/CAR vector batches produced using the RCP (Figure 9a), a detection assay was performed based on the published two-phase protocol [39,40,41]. For this, 2 × 10^5^ C8166 cells were transduced with 2.18 × 10^7^ TU of the first batch (Table 1), and a second flask was transduced with 3.28 × 10^7^ TU of the second batch. During the 21-day amplification phase, p24 levels declined from approximately 100–200 pg/mL at passage 1 (residual input LV particles) to below the limit of detection (LOD = 8 pg/mL) after passage 4. After transferring cell-free supernatant from the amplification phase to naïve C8166 cells and incubating for 7 days, no p24 signal was detectable in culture supernatants. Thus, both criteria for RCL absence—a progressive decline in p24 during amplification and p24 levels below the LOD during the indicator phase—were met. In total, over 5 × 10^7^ TU tested across two independent production batches showed no evidence of RCL, demonstrating that flavivirus-based replicons are not prone to productive recombination under laboratory conditions.

## 4. Discussion

Lentiviral vectors (LVs) are a key platform for cell and gene therapy (CGT) [43,44,45], but limited manufacturing capacity remains a major barrier to broader adoption [45]. Most current LV production relies on transient transfection with plasmids encoding HIV-1 Gag/Pol, VSV-G envelope, Tat/Rev, and the transfer vector [18,46]. This approach is widely used due to its long history, but has well-recognized limitations: high consumption of high-quality plasmid DNA and poor scalability [46,47,48,49,50].

For industrial applications, stable producer cell lines (PCLs) that enhance standardization are under development [51,52]. However, establishing stable PCLs for LV production is technically difficult because certain packaging components—particularly HIV-1 protease and VSV-G—are cytotoxic [17,53,54]. For academic research as well, alternative packaging strategies are of interest. One approach is “pseudostable cell pools”—mixed populations of transfected cells that continuously produce packaging proteins without genomic integration [55]. Replicon cell pools (RCPs), such as the system described here, bridge conventional transient transfection and classical PCLs. We explored LV production using an RNA replicon derived from yellow fever virus (a member of the genus *Orthoflavivirus*) to express Gag-Pol. The replicon retains cis-acting elements and nonstructural proteins (including RNA-dependent RNA polymerase) but lacks structural genes, enabling autonomous cytoplasmic replication without infectious particle formation [56]. Translation of replicon RNA provides high-level heterologous gene expression. Exemplary, alphavirus-derived replicons are widely used as self-amplifying RNAs for recombinant protein expression and vaccine platforms [56,57].

Although RNA replicons have rarely been used to produce LV packaging proteins, several features make them attractive: cytoplasmic replication eliminates dependence on nuclear functions; non-cytopathic variants enable prolonged expression [58,59]; replicon-driven expression is highly efficient [59]; and RCPs are easier to generate than classical PCLs because they eliminate clonal selection, shortening the development timeline [19].

Consistent with this rationale, our results indicate that LV yields from the RCP system fall within the same range as those typically obtained with conventional transient transfection in our laboratory. However, because only two independent production runs were performed (Table 1), these data should be considered preliminary. Notably, as RCP-based systems remain at an early stage, there is considerable potential for further optimisation.

Expression of wild-type HIV-1 protease (PR) was reported to be cytotoxic due to indiscriminate cleavage of host proteins [32]. Consistently, transfection with a replicon encoding wild-type PR resulted in rapid cell death within 24–48 h after puromycin selection. In contrast, the T26S mutant replicon showed no measurable cytopathic effect and preserved cell viability, as confirmed by MTT assay. The T26S substitution was mapped to the protease active site [42]. In our study, introducing the T26S mutation enabled antibiotic selection and propagation of the cell pool bearing the mutant replicon. Our Western blot analysis showed reduced processing of Pr55^gag^ to mature p24. Nevertheless, functional assays confirmed sufficient residual proteolytic activity for particle maturation and LV infectivity. These findings are consistent with those of Konvalinka et al. [42], who reported that the T26S mutation abolishes PR-mediated cleavage of host proteins without significantly affecting HIV-1 infectivity.

The rationale for developing an RCP in this study was informed by the LentiPro26 PCL described by Tomás et al. [60], which also employs the T26S-mutated Gag-Pol. In that study, the T26S mutation mitigated protease cytotoxicity and enabled constitutive Gag-Pol expression, allowing selection of high-titer producer clones such as LentiPro26-A59, which maintained titers above 10^6^ TU/mL/day. However, establishing such PCLs requires extensive clonal screening, a labor-intensive process that is more feasible in industrial settings than in academic laboratories. Our study builds on the use of the T26S protease mutant but in a different expression context: using a cytoplasmically replicating YFV replicon, we expressed the Gag-(T26S)Pol cassette to generate an RCP, providing a faster route to a producer cell pool without extensive clonal selection.

Direct sequencing of PCR products revealed no mutations in the Gag-Pol insert after ten passages of RCP cells under continuous selection. Given the polyclonal nature of the RCP and the lack of cloning, low-frequency mutants may have escaped detection. Nevertheless, sustained p24 production throughout the ten passages indicated that the majority of cells preserved functional Gag-Pol expression. This passage number was sufficient to generate 10^8^–10^9^ cells for seeding multilayer stacks, enabling LV production at scales sufficient for CAR-T manufacturing on the CliniMACS Prodigy instrument. Thus, while further characterization may be warranted for future clinical development, the RCP stability proved adequate for academic purposes.

Although the RCP described here represents a first-generation demonstrator platform in which only a subset of LV packaging proteins is expressed from the RNA replicon (other components and the LV genome are still supplied from plasmids after transfection), our results demonstrate that RCPs can maintain the balanced expression of viral proteins required for efficient LV particle assembly. Future development of this platform will focus on extending replicon-mediated expression to additional packaging components, potentially enabling fully replicon-driven LV production systems and further reducing dependence on transient transfection.

In our experiments, LV production in five-layer culture flasks yielded ~10^9^ TU. This is within the range reported for conventional transient transfection in similar culture vessels in the review of Merten et al. [17]. Similar yields have also been reported for optimized stable producer cell lines [60]. For example, this production scale is sufficient to support multiple CAR-T manufacturing runs using the CliniMACS Prodigy instrument (a cell-processing device widely used in the CAR-T field) and is adequate for the production of CAR-T cells for preclinical and early clinical studies.

The approximately twofold lower specific infectivity of RCP-produced vectors (379–472 vs. 710–907 TU/pg p24, Table 2) likely reflects impaired Gag processing due to the T26S protease mutation (Figure 2e), which increases the proportion of immature particles. Constitutive Gag-Pol expression from the replicon, combined with transient supply of Tat/Rev and envelope, may also disrupt assembly stoichiometry. Nevertheless, a single 5-layer stack run yields 4–7 × 10^8^ TU, well above the 2 × 10^8^ TU required for clinical-scale CAR-T cell product manufacturing. Furthermore, the presented packaging system holds potential for further optimization, for example by refining intergenic elements (2A, IRES, etc.) or generating RCPs with simultaneous replication of two replicons for expression of different components.

Conventional transient transfection is associated with a high cost of goods (COG), challenges in scaling the transfection process from laboratory to industrial scales, batch-to-batch variability, and the need to purify the final product from residual plasmid DNA. Therefore, both academic and industrial stakeholders are developing alternative packaging systems (PCL, RCP) to reduce COGs. Published data indeed demonstrate COG reductions, e.g., as reported for VIVEbiotech’s EvoLVcell platform [61] and the CSL Cytegrity system [62,63], as well as a decrease in process-related impurities upon adoption of fully PCL-based manufacturing. Although our RCP-produced vector showed lower specific infectivity (TU/pg p24) than conventional methods, it still achieved 77.4% CAR-positive cells using the CliniMACS Prodigy platform with the standard TCT 2.0 protocol. Thus, reduced specific infectivity does not preclude clinically relevant CAR-T manufacturing. Further improvements in transduction efficiency are possible using established enhancers (e.g., Retronectin).

No replication-competent lentivirus (RCL) was detected in 5 × 10^7^ TU tested across two independent production batches, indicating that YFV-derived replicons are not prone to productive recombination under the tested conditions. The p24 ELISA-based two-phase assay used here is appropriate for a proof-of-concept study and has been widely employed for RCL screening in research settings [39,40,41]. In this context, it is noteworthy that, unlike several other positive-sense RNA viruses, flaviviruses exhibit intrinsically low recombination rates, a property attributed to their highly organized replication compartments, which restrict template switching [30,31]. The absence of detectable RCL is therefore consistent with this lower propensity for recombination. Nevertheless, future development of this platform will require RCL testing using more stringent assays.

The replicon in this study is derived from the live-attenuated yellow fever virus vaccine strain YFV-17D, which is classified as a Risk Group 2 agent by the American Biological Safety Association [64]. Because the replicon lacks the structural genes of YFV, it cannot generate infectious viral particles, which supports the biosafety of this platform.

Several limitations of this first-generation RCP platform should be acknowledged. First, although a direct side-by-side comparison was performed between the RCP system and conventional transient transfection, the limited number of independent runs precludes definitive statistical conclusions. A larger study is planned with an improved RCP system. Second, while Sanger sequencing confirmed the absence of mutations in the Gag-Pol insert after ten passages, this method does not detect low-frequency variants. Third, our RCL assay relied on the analytical sensitivity of ELISA. Future RCL testing will require more sensitive technologies. Fourth, this first-generation RCP expresses only Gag-Pol from the replicon; other packaging components and the transfer vector still require plasmid transfection. Ongoing efforts aim to develop RCPs that additionally produce envelope protein and the vector genome from autonomously replicating RNAs.

## 5. Conclusions

This study demonstrates that a flavivirus replicon encoding HIV-1 Gag-Pol with the T26S protease mutation enables non-cytotoxic expression in replicon cell pools (RCPs). These RCPs produced lentiviral vector yields (up to ~10^9^ TU from multilayer stacks) within the same order of magnitude as conventional transient transfection, sufficient to support academic-scale CAR-T production. These findings support further development of flavivirus replicon-based packaging platforms.

## Figures and Tables

**Table 1:** Comparison of lentiviral vector production using the RCP-based system and a conventional three-plasmid transient transfection.

Process Stage	Final Volume (mL)	Concentration Factor (Fold)	Functional Titer (TU/mL) ^1^	Total Yield (TU) ^1^	Stage Yield (%) ^1^
LV packaging in Replicon Cell Pool (RCP)					
Supernatant collection	150	1×	4.20 × 10^6^ 6.06 × 10^6^	6.30 × 10^8^ 9.09 × 10^8^	100% ^2^ 100%
Concentration (TFF)	7	~21×	8.43 × 10^7^ 1.18 × 10^8^	5.90 × 10^8^ 8.23 × 10^8^	93.7% 90.6%
Purification by centrifugal pelletting	0.5	14×	8.72 × 10^8^ 1.31 × 10^9^	4.36 × 10^8^ 6.56 × 10^8^	73.8% 79.6%
Final yield	0.5	300×	8.72 × 10^8^ 1.31 × 10^9^	4.36 × 10^8^ 6.56 × 10^8^	69.2% 72.1%
Production LV using conventional packaging system					
Supernatant collection	150	1×	8.01 × 10^6^ 8.23 × 10^6^	1.20 × 10^9^ 1.23 × 10^9^	100% ^2^ 100%
Concentration (TFF)	7	~21×	1.59 × 10^8^ 1.68 × 10^8^	1.11 × 10^9^ 1.18 × 10^9^	92.4% 95.2%
Purification by centrifugal pelletting	0.5	14×	1.74 × 10^9^ 1.85 × 10^9^	8.71 × 10^8^ 9.26 × 10^8^	78.5% 78.8%
Final yield	0.5	300×	1.74 × 10^9^ 1.85 × 10^9^	8.71 × 10^8^ 9.26 × 10^8^	72.5% 75.0%

Comments: ^1^ Results from two independent experiments are shown for each packaging system. In each cell of the table, the upper value corresponds to biological repeat #1 and the lower value to biological repeat #2. ^2^ For the supernatant collection stage, the yield is arbitrarily set to 100%.

**Table 2:** Comparison of lentiviral vector specific activity (TU/pg p24) for LV preparations produced in RCP cells and in the conventional packaging system.

Packaging System ^1^	Functional Titer (TU/mL)	p24 (ng/mL)	TU/pg p24
RCP (repeat 1)	8.72 × 10^8^	2301	379
RCP (repeat 2)	1.31 × 10^9^	2775	472
Conventional (repeat 1)	1.74 × 10^9^	2451	710
Conventional (repeat 2)	1.85 × 10^9^	2040	907

Comments: ^1^ Results from two independent experiments are shown for each packaging system.

## Data Availability

Data is contained within the article or Supplementary Material.

## Supplementary Materials

The following supporting information can be downloaded at https://www.mdpi.com/article/10.3390/biology15110848/s1. Figure S1: Schematic of YFV replicons YFrep/GFP-Gag-Pol and YFrep/GFP-Gag-Pol*; Figure S2: Design of YFV replicons YFrep/GAG-POL/Pac and YFrep/GAG-POL*/Pac; Figure S3: Complete nucleotide sequence of the molecular infectious clone pYFrep/GAG-POL*/Pac; Figure S4: Flow cytometry gating strategy for counting GFP-positive cells;; Figure S5: Full membrane image of Western blot analysis of Gag processing in HEK293FT cells; Figure S6: Characterization of cell types in the CAR-T cell product obtained using the lentiviral vector LV/CAR packaged in replicon cell pools (HEK293FT-GP). Table S1: Primers used for sequencing the Gag-Pol insert in the replicon; Table S2: Representative data used to compute the limit of detection (LOD) for p24 ELISA.

## Abbreviations

The following abbreviations are used in this manuscript:

CAR	Chimeric antigen receptor
CMV	Cytomegalovirus
COG	Cost of goods
CPE	Cytopathic effect
EMCV IRES	Encephalomyocarditis virus internal ribosome entry site
GFP	Green fluorescent protein
HDV	Hepatitis delta virus
HGH	Human growth hormone
HIV	Human immunodeficiency virus
LV	Lentiviral vector
MIC	Molecular infectious clone
MOI	Multiplicity of infection
MTT	3-[4,5-dimethylthiazol-2-yl]-2,5 diphenyl tetrazolium bromide
NS	Non-structural protein
Pac	Puromycin acetyltransferase
PCLs	Packaging cell lines
PE	Phycoerythrin
PR	Protease
RCL	Replication-competent lentivirus
RCPs	Replicon cell pools
SD	Standard deviation
TFF	Tangential flow filtration
TU	Transducing units
VEEV	Venezuelan equine encephalitis virus
VLP	Virus-like particle
VSV-G	Vesicular stomatitis virus G protein
YFV	Yellow fever virus
