# Supplementary material for: Development of Replicon Cell Pools Bearing a Flavivirus RNA Replicon as a Source of HIV-1 Gag-Pol for Lentiviral Vector Production
Source: Biology (Basel). 2026 May 28;15(11):848. doi: 10.3390/biology15110848 (PMC13255789; doi:10.3390/biology15110848)
Supplement: Supplementary file 1 [file biology-15-00848-s001.zip › biology-4232255-supplementary.pdf]

Supplementary material to Development of Replicon Cell Pools Bearing a Flavivirus RNA Replicon as a Source of HIV-1 Gag-Pol for Lentiviral Vector Production

Aitolkyn Kydyrbayeva [1], Viktoriya Keyer [1], Tolganay Kulatay [1], Gulzat Zauatbayeva [1], Bakytkali Ingirbay [1], Maral Zhumabekova [1], Arman Abeev [1], Gaziza Nigmatulla [1], Alexandr V. Shustov [1,*]

[1] National Center for Biotechnology, Korgalzhin hwy 13/5, 010000, Astana, Kazakhstan
[*] Correspondence: shustov@biocenter.kz , Tel: +77024735305

A.K.	Aitolkyn Kydyrbayeva	ORCID 0009-0003-2199-5756	aitolkyn.yk@gmail.com
V.K.	Viktoriya Keyer	ORCID 0000-0001-8885-2387	keer@biocenter.kz
T.K.	Tolganay Kulatay	ORCID 0009-0004-5885-8963	kulatay@biocenter.kz
G.Z.	Gulzat Zauatbayeva	ORCID 0000-0003-1514-9302	zauatbaeva@biocenter.kz
B.I.	Bakytkali Ingirbay	ORCID 0000-0002-6915-8207	ingirbay@biocenter.kz
M.Z.	Maral Zhumabekova	ORCID 0009-0002-9532-6232	zhumabekova@biocenter.kz
A.A.	Arman Abeev	ORCID 0009-0005-3594-425X	abeev@biocenter.kz
G.M.	Gaziza Nigmatulla	ORCID 0009-0001-7868-3396	nigmatullag@gmail.com
A.V.S.	Alexandr V. Shustov	ORCID 0000-0001-9880-9382	shustov@biocenter.kz

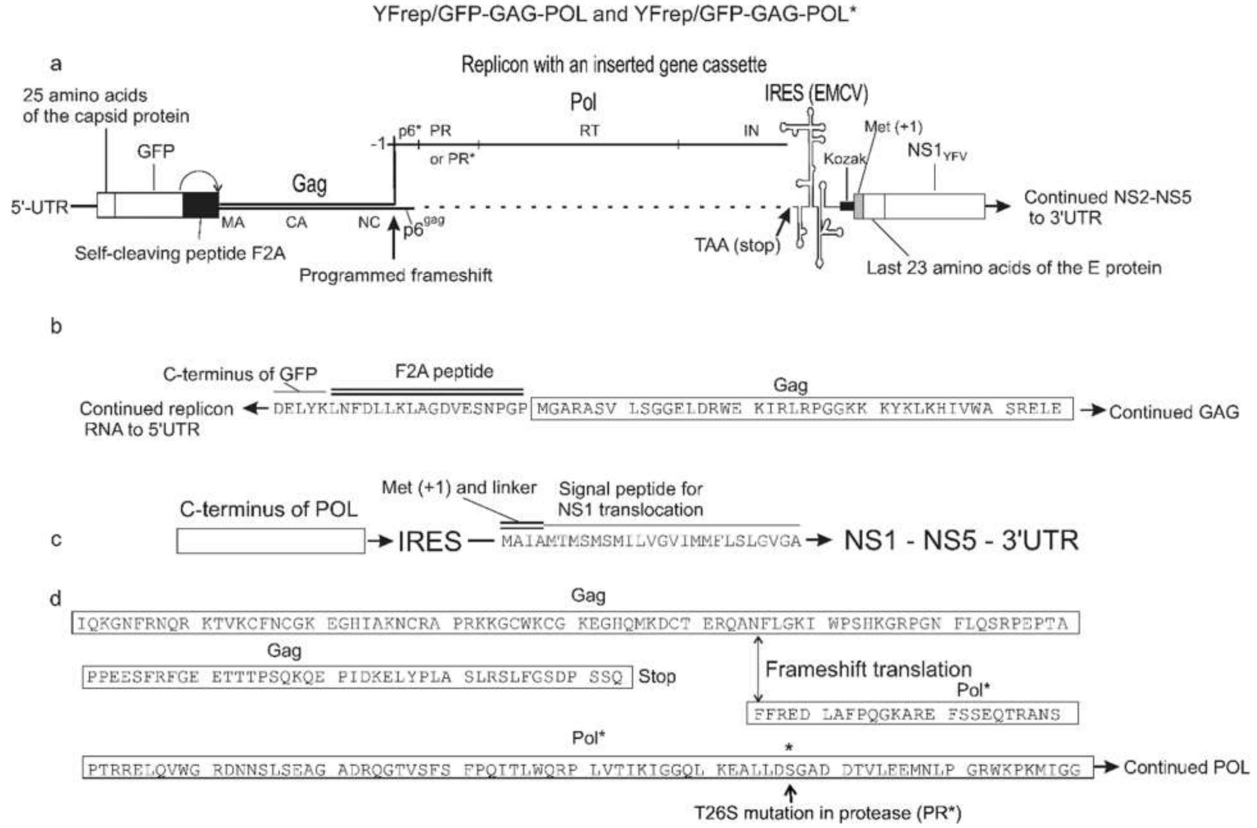

Figure S1. Schematic of the yellow fever virus (YFV) replicons YFrep/GFP-Gag-Pol and YFrep/GFP-Gag-Pol*. (**a**) Strategy for inserting a GFP-Gag-Pol cassette into YFV replicons. The 5′UTR and essential cyclization signal (25aa(n)_C) are retained. The gene cassette replaces the YFV structural genes (C-prM-E) and consists of genes encoding green fluorescent protein (GFP), foot-and-mouth disease virus self-cleaving peptide (F2A) (shown as a black rectangle with an arrow indicating the cleavage site), HIV-1 Gag-Pol, and the encephalomyocarditis virus internal ribosome entry site (EMCV IRES). The IRES directs translation of the YFV non-structural proteins NS1-NS5. The translational context includes an optimized Kozak sequence and an engineered start codon (Met+1). The HIV-1 polyprotein is depicted with its functional domains: matrix (MA), capsid (CA), nucleocapsid (NC), p6gag, p6*, protease (PR), reverse transcriptase (RT), and integrase (IN). (**b**) The amino acid sequence of a part of the protein product encoded in the first cistron of the bicistronic gene cassette. The shown segment includes the end of GFP, the F2A peptide, and the beginning of HIV-1 Gag. Translation initiates at the authentic start codon of the YFV capsid protein and proceeds through a 25-amino acid YFV capsid fragment, GFP, the F2A autoprotease, and HIV-1 Gag (enclosed in a rectangle). The HIV-1 POL gene is translated via a programmed ribosomal frameshift at the GAG/POL junction, and translation of the GAG-POL genes is terminated at the TAA stop codon, after which the EMCV IRES is positioned. (**c**) Schematic of the Pol C-terminus, IRES, and the amino acid sequence of the second (IRES-controlled) cistron translation product. The shown sequence includes the engineered start

codon and a short linker (Met-Ala-Ile-Ala), and the last 23 amino acids of the YFV envelope (E) protein, which serve as the signal peptide for NS1 translocation. (**d**) The Gag C-terminus and Pol N-terminus are shown with their reading frames enclosed in rectangles. The Gag/Pol junction contains the ribosomal frameshift site (shown with a double-headed arrow), where translation continues via a −1 frameshift. In the mutant variant Pol*, the attenuating T26S substitution is present (indicated by an upward arrow). The corresponding mutant protease (in the Pol* translation product) is labeled PR*.

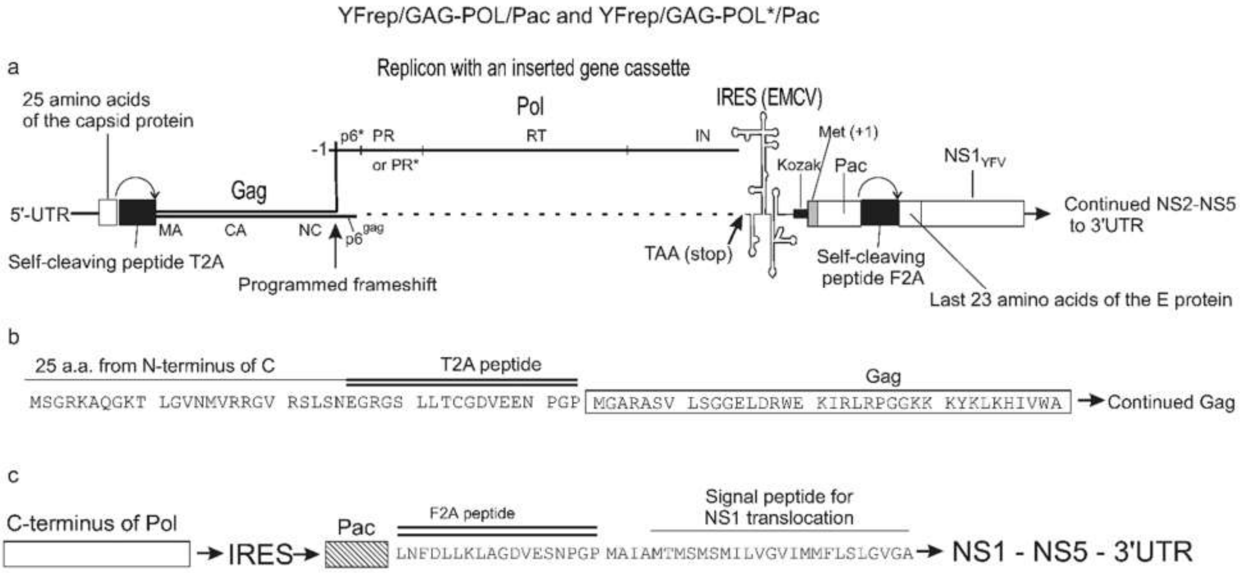

Figure S2. Design of YFV replicons YFrep/GAG-POL/Pac and YFrep/GAG-POL*/Pac.
(**a**) The structural genes (C-prM-E) are replaced by HIV-1 GAG-POL genes followed by an EMCV IRES. The IRES drives translation of a second cistron encoding puromycin acetyltransferase (Pac) and the YFV non-structural proteins NS1–NS5. The essential cyclization signal, located within a 75 nt fragment of the capsid protein gene, is retained. Self-cleaving 2A peptides (black rectangles) are placed at the junctions between the capsid fragment and HIV-1 Gag, as well as between Pac and NS1, to ensure proper separation of the heterologous and YFV proteins.
(**b**) Amino acid sequences at the beginning of the first cistron: YFV capsid fragment (first 25 amino acids of C), the *Thosea asigna* virus self-cleaving peptide (T2A), and the N-terminus of HIV-1 Gag.
(**c**) Schematic of the junction between the heterologous cassette and the non-structural genes in the YFV replicon. The second (IRES-controlled) cistron encodes puromycin acetyltransferase (Pac), followed by the foot-and-mouth disease virus self-cleaving peptide (F2A) and the YFV non-structural proteins NS1–NS5. The amino acid sequence shown includes the F2A, an engineered start codon with a short linker (Met-Ala-Ile-Ala), and the last 23 amino acids of the YFV envelope (E) protein, which serve as the signal peptide for NS1 translocation.

```
LOCUS       YFrep_GAG-POL_Pac       19495 bp    DNA     circular      5-MAY-2026
SOURCE
  ORGANISM
COMMENT     This file is created by Vector NTI
            http://www.invitrogen.com/
COMMENT     VNTDATE|-16482972|
COMMENT     VNTDBDATE|-16482976|
COMMENT     LSOWNER|
COMMENT     VNTNAME|YFrep_GAG-POL_Pac|
COMMENT     VNTAUTHORNAME|Demo User|
COMMENT     Vector_NTI_Display_Data_(Do_Not_Edit!)
COMMENT     (SXF
COMMENT      (CGexDoc "YFrep_GAG-POL_Pac" 0 19495
COMMENT       (CDBMol 0 0 1 1 1 0 0 1 0 "" "" 0 0 0 0 (CObList) (CObList) (CObList)
COMMENT        (CObList) -1 "")
COMMENT       (CDocSetData 1 1 0 0 0 0 "MAIN" 1 1 1 1 0 0 1 1 0 1 10 10 4294967295 50 0
COMMENT        1 0 (CHomObj 0 0 0 3 75) (CWordArray) (CWordArray) (CStringList)
COMMENT        (CStringList "atg" "gtg") (CStringList "taa" "tga" "tag") (CObList) 1
COMMENT        "{(0,1),2}" 0 0 "" 0 4294967295 0 0 0 0 0 0 0 "MAIN" 0 0 30 0
COMMENT        (CProteinMotifSearchObject 70 20 1 1 1 1 0 0 1 0 0 0 0 0))
COMMENT       (CMolPar 0 0 0 0 0 1 19495 0 0 0 0 0 0 0 0) (CStringList) (CStringList)
COMMENT       (CObList) (COAPar 25 250 50 0 6 4 3 7) (COAPar 25 250 50 0 6 4 3 7)
COMMENT       (COAPar 25 250 50 0 6 4 3 7) (CObList)
COMMENT       (CObList
COMMENT        #0=(CFSignal (CObList) "HDV antigenomic ribozyme" 21 0 0 14286 14369 0
COMMENT            (CStringList) (CStringList) 1 1 1 1 "14276..14359")
COMMENT        #1=(CFSignal (CObList) "SV40 ori" 33 0 0 15448 15750 0 (CStringList)
COMMENT            (CStringList) 1 1 1 1 "15438..15740")
COMMENT        #2=(CFSignal (CObList) "Neomycin resistance (Neo)" 4 0 0 15817 16611 0
COMMENT            (CStringList) (CStringList) 1 1 1 1 "15807..16601")
COMMENT        #3=(CFSignal (CObList) "PolyA from human growth hormone gene" 25 0 0
COMMENT            14778 15363 0 (CStringList) (CStringList) 1 1 1 1 "14768..15353")
COMMENT        #4=(CFSignal (CObList) "Ampicillin resistance (AmpR)" 4 0 1 17889 18749
COMMENT            0 (CStringList) (CStringList) 1 1 1 1 "complement(961..1821)")
COMMENT        #5=(CFSignal (CObList) "Pac (puromycin resistance)" 4 0 0 5141 5740 0
COMMENT            (CStringList) (CStringList) 1 1 1 1 "")
COMMENT        #6=(CFSignal (CObList) "POL" 4 0 0 1543 4551 0 (CStringList)
COMMENT            (CStringList) 1 1 1 1 "")
COMMENT        #7=(CFSignal (CObList) "GAG" 4 0 0 248 1747 0 (CStringList)
COMMENT            (CStringList) 1 1 1 1 "")
COMMENT        #8=(CFSignal (CObList) "Signal peptide for NS1 translocation" 4 0 0 5807
COMMENT            5875 0 (CStringList) (CStringList) 1 1 1 1 "")
COMMENT        #9=(CFSignal (CObList) "T2A self-cleaving peptide" 4 0 0 194 247 0
COMMENT            (CStringList) (CStringList) 1 1 1 1 "")
COMMENT        #10=(CFSignal (CObList) "IRES (EMCV)" 21 0 0 4559 5126 0 (CStringList)
COMMENT             (CStringList) 1 1 1 1 "")
COMMENT        #11=(CFSignal (CObList) "NS1" 4 0 0 5876 7102 0 (CStringList)
COMMENT             (CStringList) 1 1 1 1 "3300..4526")
COMMENT        #12=(CFSignal (CObList) "NS5" 4 0 0 11060 13777 0 (CStringList)
COMMENT             (CStringList) 1 1 1 1 "8484..11201")
COMMENT        #13=(CFSignal (CObList) "3'UTR" 50 0 0 13778 14285 0 (CStringList)
COMMENT             (CStringList) 1 1 1 1 "11202..11709")
COMMENT        #14=(CFSignal (CObList) "25 aa from N-end capsid" 4 0 0 119 193 0
COMMENT             (CStringList) (CStringList) 1 1 1 1 "120..194")
COMMENT        #15=(CFSignal (CObList) "5'UTR" 52 0 0 1 118 0 (CStringList)
COMMENT             (CStringList) 1 1 1 1 "2..119")
COMMENT        #16=(CFSignal (CObList) "NS2" 4 0 0 7103 7993 0 (CStringList)
COMMENT             (CStringList) 1 1 1 1 "4527..5417")
COMMENT        #17=(CFSignal (CObList) "NS3" 4 0 0 7994 9862 0 (CStringList)
COMMENT             (CStringList) 1 1 1 1 "5418..7286")
COMMENT        #18=(CFSignal (CObList) "NS4" 4 0 0 9863 11059 0 (CStringList)
COMMENT             (CStringList) 1 1 1 1 "7287..8483")
COMMENT        #19=(CFSignal (CObList) "FMDV 2A self-cleaving peptide" 4 0 0 5744 5794
```

```
COMMENT                             (CWidget 52 (7 52 0) 1 2 0 0 Nil -395 100)
COMMENT                             (CObjectList
COMMENT                              #206=(CScratch
COMMENT                                    (CWidget 0 (3 #15# 0) 1 2 0 0 #120# 0 100)
COMMENT                                    (LOGPEN 0 6 6723840) 8 1 6.27319 1.9 0.082322
COMMENT                                    1)
COMMENT                              #207=(CLabel
COMMENT                                    (CWidget 0 (0 0) 1 2 0 0 #114# 1953720691 100)
COMMENT                                    (LOGPEN 0 0 0) 1
COMMENT                                    (LOGFONT 46 17 0 0 700 0 0 0 0 3 2 1 34
COMMENT                                     "Arial") 1.74233 0.666667 0 "5'UTR" "@N" 1
COMMENT                                    2.31687 13.9386 0 -10 2.52262 1.17389 #206#))
COMMENT                             (CObjectList))) (CObjectList))
COMMENT                #208=(CGroupWidget (CWidget 14 (16 0) 1 2 0 0 Nil 1638 100)
COMMENT                      (CObjectList) (CObjectList))
COMMENT                #209=(CGroupWidget (CWidget 11 (0 0) 1 2 0 0 Nil -325 100)
COMMENT                      (CObjectList) (CObjectList))
COMMENT                #210=(CGroupWidget (CWidget 12 (0 0) 1 2 0 0 Nil -185 100)
COMMENT                      (CObjectList) (CObjectList))) (CObjectList)))
COMMENT       (CSeqView 10 10 (CObjectList) (CObList) 1 (CObList)) (CObList) 0
COMMENT       (CStringList) 0 0 (CObList)))
```


```
FEATURES             Location/Qualifiers
     misc_feature    14286..14369
                     /vntifkey="21"
                     /label=HDV\antigenomic\ribozyme
     rep_origin      15448..15750
                     /vntifkey="33"
                     /label=SV40\ori
     CDS             15817..16611
                     /vntifkey="4"
                     /label=Neomycin\resistance\(Neo)
     polyA_signal    14778..15363
                     /vntifkey="25"
                     /label=PolyA\from\human\growth\hormone\gene
     CDS             complement(17889..18749)
                     /vntifkey="4"
                     /label=Ampicillin\resistance\(AmpR)
     CDS             5141..5740
                     /vntifkey="4"
                     /label=Pac\(puromycin\resistance)
     CDS             1543..4551
                     /vntifkey="4"
                     /label=POL
     CDS             248..1747
                     /vntifkey="4"
                     /label=GAG
     CDS             5807..5875
                     /vntifkey="4"
                     /label=Signal\peptide\for\NS1\translocation
     CDS             194..247
                     /vntifkey="4"
                     /label=T2A\self-cleaving\peptide
     misc_feature    4559..5126
                     /vntifkey="21"
                     /label=IRES\(EMCV)
     CDS             5876..7102
                     /vntifkey="4"
                     /label=NS1
                     /note="Soluble Complement-Fixing Antigen"
     CDS             11060..13777
                     /vntifkey="4"
                     /label=NS5
     3'UTR           13778..14285
```

```
                     /vntifkey="50"
                     /label=3'UTR
     CDS             119..193
                     /vntifkey="4"
                     /label=25\aa\from\N-end\capsid
     5'UTR           1..118
                     /vntifkey="52"
                     /label=5'UTR
     CDS             7103..7993
                     /vntifkey="4"
                     /label=NS2
     CDS             7994..9862
                     /vntifkey="4"
                     /label=NS3
     CDS             9863..11059
                     /vntifkey="4"
                     /label=NS4
     CDS             5744..5794
                     /vntifkey="4"
                     /label=FMDV\2A\self-cleaving\peptide
     promoter        18899..19495
                     /vntifkey="29"
                     /label=CMV\promoter
BASE COUNT     5481 a      4389 c      5209 g      4416 t
ORIGIN
        1 agtaaatcct gtgtgctaat tgaggtgcat tggtctgcaa atcgagttgc taggcaataa
       61 acacatttgg attaatttta atcgttcgtt gagcgattag cagagaactg accagaacat
      121 gtctggtcgt aaagctcagg gaaaaaccct gggcgtcaat atggtacgac gaggagttcg
      181 ctccttgtca aacgagggca ggggaagctt gctgacatgt ggcgacgtgg aggaaaaccc
      241 tggacccatg ggtgcgagag cgtcagtatt aagcggggga gaattagatc gatgggaaaa
      301 aattcggtta aggccagggg gaaagaaaaa atataaatta aaacatatag tatgggcaag
      361 cagggagcta gaacgattcg cagttaatcc tggcctgtta gaaacatcag aaggctgtag
      421 acaaatactg ggacagctac aaccatccct tcagacagga tcagaagaac ttagatcatt
      481 atataataca gtagcaaccc tctattgtgt gcatcaaagg atagagataa aagacaccaa
      541 ggaagcttta gacaagatag aggaagagca aaacaaaagt aagaaaaaag cacagcaagc
      601 agcagctgac acaggacaca gcaatcaggt cagccaaaat taccctatag tgcagaacat
      661 ccaggggcaa atggtacatc aggccatatc acctagaact ttaaatgcat gggtaaaagt
      721 agtagaagag aaggctttca gcccagaagt gatacccatg ttttcagcat tatcagaagg
      781 agccacccca caagatttaa acaccatgct aaacacagtg gggggacatc aagcagccat
      841 gcaaatgtta aaagagacca tcaatgagga agctgcagaa tgggatagag tgcatccagt
      901 gcatgcaggg cctattgcac caggccagat gagagaacca aggggaagtg acatagcagg
      961 aactactagt acccttcagg aacaaatagg atggatgaca cataatccac ctatcccagt
     1021 aggagaaatc tataaaagat ggataatcct gggattaaat aaaatagtaa gaatgtatag
     1081 ccctaccagc attctggaca taagacaagg accaaaggaa ccctttagag actatgtaga
     1141 ccgattctat aaaactctaa gagccgagca agcttcacaa gaggtaaaaa attggatgac
     1201 agaaaccttg ttggtccaaa atgcgaaccc agattgtaag actattttaa aagcattggg
     1261 accaggagcg acactagaag aaatgatgac agcatgtcag ggagtggggg gacccggcca
     1321 taaagcaaga gttttggctg aagcaatgag ccaagtaaca aatccagcta ccataatgat
     1381 acagaaaggc aattttagga accaaagaaa gactgttaag tgtttcaatt gtggcaaaga
     1441 agggcacata gccaaaaatt gcagggcccc taggaaaaag ggctgttgga aatgtggaaa
     1501 ggaaggacac caaatgaaag attgtactga gagacaggct aatttttag ggaagatctg
     1561 gccttcccac aagggaaggc cagggaattt tcttcagagc agaccagagc caacagcccc
     1621 accagaagag agcttcaggt ttggggaaga gacaacaact ccctctcaga agcaggagcc
     1681 gatagacaag gaactgtatc ctttagcttc cctcagatca ctctttggca gcgacccctc
     1741 gtcacaataa agataggggg gcaattaaag gaagctctat tagatacagg agcagatgat
     1801 acagtattag aagaaatgaa tttgccagga agatggaaac caaaaatgat agggggaatt
     1861 ggaggtttta tcaaagtaag acagtatgat cagatactca tagaaatctg cggacataaa
     1921 gctataggta cagtattagt aggacctaca cctgtcaaca taattggaag aaatctgttg
     1981 actcagattg gctgcacttt aaattttccc attagtccta ttgagactgt accagtaaaa
     2041 ttaaagccag gaatggatgg cccaaaagtt aaacaatggc cattgacaga agaaaaaata
     2101 aaagcattag tagaaatttg tacagaaatg gaaaaggaag gaaaaatttc aaaaattggg
     2161 cctgaaaatc catacaatac tccagtattt gccataaaga aaaaagacag tactaaatgg
     2221 agaaaattag tagatttcag agaacttaat aagagaactc aagatttctg ggaagttcaa
```

2281 ttaggaatac cacatcctgc agggttaaaa cagaaaaaat cagtaacagt actggatgtg
2341 ggcgatgcat atttttcagt tcccttagat aaagacttca ggaagtatac tgcatttacc
2401 atacctagta taaacaatga gacaccaggg attagatatc agtacaatgt gcttccacag
2461 ggatggaaag gatcaccagc aatattccag tgtagcatga caaaaatctt agagcctttt
2521 agaaaacaaa atccagacat agtcatctat caatacatgg atgatttgta tgtaggatct
2581 gacttagaaa tagggcagca tagaacaaaa atagaggaac tgagacaaca tctgttgagg
2641 tggggattta ccacaccaga caaaaaacat cagaaagaac ctccattcct ttggatgggt
2701 tatgaactcc atcctgataa atggacagta cagcctatag tgctgccaga aaaggacagc
2761 tggactgtca atgacataca gaaattagtg ggaaaattga attgggcaag tcagatttat
2821 gcagggatta aagtaaggca attatgtaaa cttcttaggg gaaccaaagc actaacagaa
2881 gtagtaccac taacagaaga agcagagcta gaactggcag aaaacaggga gattctaaaa
2941 gaaccggtac atggagtgta ttatgaccca tcaaaagact taatagcaga aatacagaag
3001 caggggcaag gccaatggac atatcaaatt tatcaagagc catttaaaaa tctgaaaaca
3061 ggaaagtatg caagaatgaa gggtgcccac actaatgatg tgaaacaatt aacagaggca
3121 gtacaaaaaa tagccacaga aagcatagta atatggggaa agactcctaa atttaaatta
3181 cccatacaaa aggaaacatg ggaagcatgg tggacagagt attggcaagc cacctggatt
3241 cctgagtggg agtttgtcaa tacccctccc ttagtgaagt tatggtacca gttagagaaa
3301 gaacccataa taggagcaga aactttctat gtagatgggg cagccaatag ggaaactaaa
3361 ttaggaaaag caggatatgt aactgacaga ggaagacaaa aagttgtccc cctaacggac
3421 acaacaaatc agaagactga gttacaagca attcatctag ctttgcagga ttcgggatta
3481 gaagtaaaca tagtgacaga ctcacaatat gcattgggaa tcattcaagc acaaccagat
3541 aagagtgaat cagagttagt cagtcaaata atagagcagt taataaaaaa ggaaaaagtc
3601 tacctggcat gggtaccagc acacaaagga attggaggaa atgaacaagt agataaattg
3661 gtcagtgctg gaatcaggaa agtactattt ttagatggaa tagataaggc ccaagaagaa
3721 catgagaaat atcacagtaa ttggagagca atggctagtg attttaacct accacctgta
3781 gtagcaaaag aaatagtagc cagctgtgat aaatgtcagc taaaagggga agccatgcat
3841 ggacaagtag actgtagccc aggaatatgg cagctagatt gtacacattt agaaggaaaa
3901 gttatcttgg tagcagttca tgtagccagt ggatatatag aagcagaagt aattccagca
3961 gagacagggc aagaaacagc atacttcctc ttaaaattag caggaagatg gccagtaaaa
4021 acagtacata cagacaatgg cagcaatttc accagtacta cagttaaggc cgcctgttgg
4081 tgggcgggga tcaagcagga atttggcatt ccctacaatc cccaaagtca aggagtaata
4141 gaatctatga ataaagaatt aaagaaaatt ataggacagg taagagatca ggctgaacat
4201 cttaagacag cagtacaaat ggcagtattc atccacaatt ttaaaagaaa aggggggatt
4261 ggggggtaca gtgcagggga aagaatagta gacataatag caacagacat acaaactaaa
4321 gaattacaaa aacaaattac aaaaattcaa aattttcggg tttattacag ggacagcaga
4381 gatccagttt ggaaaggacc agcaaagctc ctctggaaag gtgaaggggc agtagtaata
4441 caagataata gtgacataaa agtagtgcca agaagaaaag caaagatcat cagggattat
4501 ggaaaacaga tggcaggtga tgattgtgtg gcaagtagac aggatgagga ttaagaattc
4561 cgcccccccc ccctaacgtt actggccgaa gccgcttgga ataaggccgg tgtgcgtttg
4621 tctatatgtt attttccacc atattgccgt cttttggcaa tgtgagggcc cggaaacctg
4681 gccctgtctt cttgacgagc attcctaggg gtctttcccc tctcgccaaa ggaatgcaag
4741 gtctgttgaa tgtcgtgaag gaagcagttc ctctggaagc ttcttgaaga caaacaacgt
4801 ctgtagcgac cctttgcagg cagcggaacc ccccacctgg cgacaggtgc ctctgcggcc
4861 aaaagccacg tgtataagat acacctgcaa aggcggcaca accccagtgc cacgttgtga
4921 gttggatagt tgtggaaaga gtcaaatggc tctcctcaag cgtattcaac aaggggctga
4981 aggatgccca gaaggtaccc cattgtatgg gatctgatct ggggcctcgg tgcacatgct
5041 ttacatgtgt ttagtcgagg ttaaaaaaac gtctaggccc cccgaaccac ggggacgtgg
5101 ttttcctttg aaaaacacga tgataactcg agccgccacc atgaccgagt acaagcccac
5161 ggtgcgcctc gccacccgcg acgacgtccc cagggccgta cgcaccctcg ccgccgcgtt
5221 cgccgactac cccgccacgc gccacaccgt cgatccggac cgccacatcg agcgggtcac
5281 cgagctgcaa gaactcttcc tcacgcgcgt cgggctcgac atcggcaagg tgtgggtcgc
5341 ggacgacggc gccgcggtgg cggtctggac cacgccggag agcgtcgaag cgggggcggt
5401 gttcgccgag atcggcccgc gcatggccga gttgagcggt tcccggctgg ccgcgcagca
5461 acagatggaa ggcctcctgg cgccgcaccg gcccaaggag cccgcgtggt tcctggccac
5521 cgtcggcgtc tcgcccgacc accagggcaa gggtctgggc agcgccgtcg tgctccccgg
5581 agtggaggcg gccgagcgcg ccggggtgcc cgccttcctg gagacctccg cgccccgcaa
5641 cctccccttc tacgagcggc tcggcttcac cgtcaccgcc gacgtcgagg tgcccgaagg
5701 accgcgcacc tggtgcatga cccgcaagcc cggtgccaag cttaacttcg acctgctgaa
5761 gctcgccggc gacgtggaga gcaaccctgg ccctatggcg atcgccatga caatgtccat
5821 gagcatgatc ttggtaggag tgatcatgat gtttttgtct ctaggagttg ggcggatca
5881 aggatgcgcc atcaactttg gcaagagaga gctcaagtgc ggagatggta tcttcatatt
5941 tagagactct gatgactggc tgaacaagta ctcatactat ccagaagatc ctgtgaagct
6001 tgcatcaata gtgaaagcct cttttgaaga agggaagtgt ggcctaaatt cagttgactc

6061 ccttgagcat gagatgtgga gaagcagggc agatgagatc aatgccattt ttgaggaaaa
6121 cgaggtggac atttctgttg tcgtgcagga tccaaagaat gtttaccaga gaggaactca
6181 tccattttcc agaattcggg atggtctgca gtatggttgg aagacttggg gtaagaacct
6241 tgtgttctcc ccagggagga agaatggaag cttcatcata gatggaaagt ccaggaaaga
6301 atgcccgttt tcaaaccggg tctggaattc tttccagata gaggagtttg ggacgggagt
6361 gttcaccaca cgcgtgtaca tggacgcagt ctttgaatac accatagact gcgatggatc
6421 tatcttgggt gcagcggtga acggaaaaaa gagtgcccat ggctctccaa cattttggat
6481 gggaagtcat gaagtaaatg ggacatggat gatccacacc ttggaggcat tagattacaa
6541 ggagtgtgag tggccactga cacatacgat tggaacatca gttgaagaga gtgaaatgtt
6601 catgccgaga tcaatcggag gcccagttag ctctcacaat catatccctg gatacaaggt
6661 tcagacgaac ggaccttgga tgcaggtacc actagaagtg aagagagaag cttgcccagg
6721 gactagcgtg atcattgatg gcaactgtga tggacgggga aaatcaacca gatccaccac
6781 ggatagcggg aaagttattc ctgaatggtg ttgccgctcc tgcacaatgc cgcctgtgag
6841 cttccatggt agtgatgggt gttggtatcc catggaaatt aggccaagga aaacgcatga
6901 aagccatctg gtgcgctcct gggttacagc tggagaaata catgctgtcc cttttggttt
6961 ggtgagcatg atgatagcaa tggaagtggt cctaaggaaa agacagggac caaagcaaat
7021 gttggttgga ggagtagtgc tcttgggagc aatgctggtc gggcaagtaa ctctccttga
7081 tttgctgaaa ctcacagtgg ctgtgggatt gcatttccat gagatgaaca atggaggaga
7141 cgccatgtat atggcgttga ttgctgcctt ttcaatcaga ccagggctgc tcatcggctt
7201 tgggctcagg accctatgga gccctcggga acgccttgtg ctgaccctag gagcagccat
7261 ggtggagatt gccttgggtg gcgtgatggg cggcctgtgg aagtatctaa atgcagtttc
7321 tctctgcatc ctgacaataa atgctgttgc ttctaggaaa gcatcaaata ccatcttgcc
7381 cctcatggct ctgttgacac ctgtcactat ggctgaggtg agacttgccg caatgttctt
7441 ttgtgccgtg gttatcatag gggtccttca ccagaatttc aaggacacct ccatgcagaa
7501 gactatacct ctggtggccc tcacactcac atcttacctg ggcttgacac aacctttttt
7561 gggcctgtgt gcatttctgg caacccgcat atttgggcga aggagtatcc cagtgaatga
7621 ggcactcgca gcagctggtc tagtgggagt gctggcagga ctggcttttc aggagatgga
7681 gaacttcctt ggtccgattg cagttggagg actcctgatg atgctggtta gcgtggctgg
7741 gagggtggat gggctagagc tcaagaagct tggtgaagtt tcatgggaag aggaggcgga
7801 gatcagcggg agttccgccc gctatgatgt ggcactcagt gaacaagggg agttcaagct
7861 gctttctgaa gagaaagtgc catgggacca ggttgtgatg acctcgctgg ccttggttgg
7921 ggctgccctc catccatttg ctcttctgct ggtccttgct gggtggctgt ttcatgtcag
7981 gggagctagg agaagtgggg atgtcttgtg ggatattccc actcctaaga tcatcgagga
8041 atgtgaacat ctggaggatg ggatttatgg catattccag tcaaccttct tgggggcctc
8101 ccagcgagga gtgggagtgg cacagggagg ggtgttccac acaatgtggc atgtcacaag
8161 aggagctttc cttgtcagga atggcaagaa gttgattcca tcttgggctt cagtaaagga
8221 agaccttgtc gcctatggtg gctcatggaa gttggaaggc agatgggatg gagaggaaga
8281 ggtccagttg atcgcggctg ttccaggaaa gaacgtggtc aacgtccaga caaaaccgag
8341 cttgttcaaa gtgaggaatg gggagaaat cggggctgtc gctcttgact atccgagtgg
8401 cacttcagga tctcctattg ttaacaggaa cggagaggtg attgggctgt acggcaatgg
8461 catccttgtc ggtgacaact ccttcgtgtc cgccatatcc cagactgagg tgaaggaaga
8521 aggaaaggag gagctccaag agatcccgac aatgctaaag aaaggaatga caactgtcct
8581 tgattttcat cctggagctg ggaagacaag acgtttcctc ccacagatct tggccgagtg
8641 cgcacggaga cgcttgcgca ctcttgtgtt ggcccccacc agggttgttc tttctgaaat
8701 gaaggaggct tttcacggcc tggacgtgaa attccacaca caggcttttt ccgctcacgg
8761 cagcgggaga gaagtcattg atgccatgtg ccatgccacc ctaacttaca ggatgttgga
8821 accaactagg gttgttaact gggaagtgat cattatggat gaagcccatt ttttggatcc
8881 agctagcata gccgctagag gttgggcagc gcacagagct agggcaaatg aaagtgcaac
8941 aatcttgatg acagccacac cgcctgggac tagtgatgaa tttccacatt caaatggtga
9001 aatagaagat gttcaaacgg acatacccag tgagccctgg aacacagggc atgactggat
9061 cctagctgac aaaaggccca cggcatggtt ccttccatcc atcagagctg caaatgtcat
9121 ggctgcctct ttgcgtaagg ctggaaagag tgtggtggtc ctgaacagga aaacctttga
9181 gagagaatac ccacgataa agcagaagaa acctgacttt atattggcca ctgacatagc
9241 tgaaatggga gccaaccttt gcgtggagcg agtgctggat tgcaggacgg cttttaagcc
9301 tgtgcttgtg gatgaaggga ggaaggtggc aataaaaggg ccacttcgta tctccgcatc
9361 ctctgctgct caaaggaggg ggcgcattgg gagaaatccc aacagagatg gagactcata
9421 ctactattct gagcctacaa gtgaaaataa tgcccaccac gtctgctggt tggaggcctc
9481 aatgctcttg gacaacatgg aggtgagggg tggaatggtc gccccactct atggcgttga
9541 aggaactaaa acaccagttt cccctggtga aatgagactg agggatgacc agaggaaagt
9601 cttcagagaa ctagtgagga attgtgacct gcccgtttgg ctttcgtggc aagtggccaa
9661 ggctggtttg aagacgaatg atcgtaagtg gtgttttgaa ggccctgagg aacatgagat
9721 cttgaatgac agcggtgaaa cagtgaagtg cagggctcct ggaggagcaa agaagcctct
9781 gcgcccaagg tggtgtgatg aaagggtgtc atctgaccag agtgcgctgt ctgaatttat

9841 taagtttgct gaaggtagga ggggagctgc tgaagtgcta gttgtgctga gtgaactccc
9901 tgatttcctg gctaaaaaag gtggagaggc aatggatacc atcagtgtgt tcctccactc
9961 tgaggaaggc tctagggctt accgcaatgc actatcaatg atgcctgagg caatgacaat
10021 agtcatgctg tttatactgg ctggactact gacatcggga atggtcatct ttttcatgtc
10081 tcccaaaggc atcagtagaa tgtctatggc gatgggcaca atggccggct gtggatatct
10141 catgttcctt ggaggcgtca aacccactca catctcctat gtcatgctca tattctttgt
10201 cctgatggtg gttgtgatcc ccgagccagg gcaacaaagg tccatccaag acaaccaagt
10261 ggcatacctc attattggca tcctgacgct ggtttcagcg gtggcagcca acgagctagg
10321 catgctggag aaaaccaaag aggacctctt tgggaagaag aacttaattc catctagtgc
10381 ttcaccctgg agttggccgg atcttgacct gaagccagga gctgcctgga cagtgtacgt
10441 tggcattgtt acaatgctct ctccaatgtt gcaccactgg atcaaagtcg aatatggcaa
10501 cctgtctctg tctggaatag cccagtcagc ctcagtcctt tctttcatgg acaaggggat
10561 accattcatg aagatgaata tctcggtcat aatgctgctg gtcagtggct ggaattcaat
10621 aacagtgatg cctctgctct gtggcatagg gtgcgccatg ctccactggt ctctcatttt
10681 acctggaatc aaagcgcagc agtcaaagct tgcacagaga agggtgttcc atggcgttgc
10741 cgagaaccct gtggttgatg ggaatccaac agttgacatt gaggaagctc ctgaaatgcc
10801 tgccctttat gagaagaaac tggctctata tctccttctt gctctcagcc tagcttctgt
10861 tgccatgtgc agaacgccct tttcattggc tgaaggcatt gtcctagcat cagctgcctt
10921 agggccgctc atagagggaa acaccagcct tctttggaat ggacccatgg ctgtctccat
10981 gacaggagtc atgaggggga atcactatgc ttttgtggga gtcatgtaca atctatggaa
11041 gatgaaaact ggacgccggg ggagcgcgaa tggaaaaact ttgggtgaag tctggaagag
11101 ggaactgaat ctgttggaca agcgacagtt tgagttgtat aaaaggaccg acattgtgga
11161 ggtggatcgt gatacggcac gcaggcattt ggccgaaggg aaggtggaca ccggggtggc
11221 ggtctccagg gggaccgcaa agttaaggtg gttccatgag cgtggctatg tcaagctgga
11281 aggtagggtg attgacctgg ggtgtggccg cggaggctgg tgttactacg ctgctgcgca
11341 aaaggaagtg agtggggtca aaggatttac tcttggaaga gacggccatg agaaacccat
11401 gaatgtgcaa agtctgggat ggaacatcat caccttcaag gacaaaactg atatccaccg
11461 cctagaacca gtgaaatgtg acaccctttt gtgtgacatt ggagagtcat catcgtcatc
11521 ggtcacagag ggggaaagga ccgtgagagt tcttgatact gtagaaaaat ggctggcttg
11581 tggggttgac aacttctgtg tgaaggtgtt agctccatac atgccagatg ttcttgagaa
11641 actggaattg ctccaaagga ggtttggcgg aacagtgatc aggaaccctc tctccaggaa
11701 ttccactcat gaaatgtact acgtgtctgg agcccgcagc aatgtcacat ttactgtgaa
11761 ccaaacatcc cgcctcctga tgaggagaat gaggcgtcca actggaaaag tgaccctgga
11821 ggctgacgtc atcctcccaa ttgggacacg cagtgttgag acagacaagg gacccctgga
11881 caaagaggcc atagaagaaa gggttgagag gataaaatct gagtacatga cctcttggtt
11941 ttatgacaat gacaacccct acaggacctg gcactactgt ggctcctatg tcacaaaaac
12001 ctcaggaagt gcggcgagca tggtaaatgg tgttattaaa attctgacat atccatggga
12061 caggatagag gaggtcacaa gaatggcaat gactgacaca acccctttg gacagcaaag
12121 agtgtttaaa gaaaaagttg acaccagagc aaaggatcca ccagcgggaa ctaggaagat
12181 catgaaagtt gtcaacaggt ggctgttccg ccacctggcc agagaaaaga accccagact
12241 gtgcacaaag gaagaattta ttgcaaaagt ccgaagtcat gcagccattg gagcttacct
12301 ggaagaacaa gaacagtgga agactgccaa tgaggctgtc caagacccaa agttctggga
12361 actggtggat gaagaaagga agctgcacca acaaggcagg tgtcggactt gtgtgtacaa
12421 catgatgggg aaaagagaga agaagctgtc agagtttggg aaagcaaagg gaagccgtgc
12481 catatggtat atgtggctgg gagcgcggta tcttgagttt gaggccctgg gattcctgaa
12541 tgaggaccat tgggcttcca gggaaaactc aggaggagga gtggaaggca ttggcttaca
12601 atacctagga tatgtgatca gagacctggc tgcaatggat ggtggtggat tctacgcgga
12661 tgacaccgct ggatgggaca cgcgcatcac agaggcagac cttgatgatg aacaggagat
12721 cttgaactac atgagcccac atcacaaaaa actggcacaa gcagtgatgg aaatgacata
12781 caagaacaaa gtggtgaaag tgttgagacc agccccagga gggaaagcct acatggatgt
12841 cataagtcga cgagaccaga gaggatccgg gcaggtagtg acttatgctc tgaacaccat
12901 caccaacttg aaagtccaat tgatcagaat ggcagaagca gagatggtga tacatcacca
12961 acatgttcaa gattgtgatg aatcagttct gaccaggctg gaggcatggc tcactgagca
13021 cggatgtgac agactgaaga ggatggcggt gagtggagac gactgtgtgg tccggcccat
13081 cgatgacagg ttcggcctgg ccctgtccca tctcaacgcc atgtccaagg ttagaaagga
13141 catatctgaa tggcagccat caaaagggtg gaatgattgg gagaatgtgc ccttctgttc
13201 ccaccacttc catgaactac agctgaagga tggcaggagg attgtggtgc cttgccgaga
13261 acaggacgag ctcattggga gaggaagggt gtctccagga aacggctgga tgatcaagga
13321 aacagcttgc ctcagcaaag cctatgccaa catgtggtca ctgatgtatt ttcacaaaag
13381 ggacatgagg ctactgtcat tggctgtttc ctcagctgtt cccacctcat gggttccaca
13441 aggacgcaca acatggtcga ttcatgggaa aggggagtgg atgaccacgg aagacatgct
13501 tgaggtgtgg aacagagtat ggataaccaa caacccacac atgcaggaca agacaatggt
13561 gaaaaaatgg agagatgtcc cttatctaac caagagacaa gacaagctgt gcggatcact

13621 gattggaatg accaataggg ccacctgggc ctcccacatc catttagtca tccatcgtat
13681 ccgaacgctg attggacagg agaaatacac tgactaccta acagtcatgg acaggtattc
13741 tgtggatgct gacctgcaac tgggtgagct tatctgaaac accatctaac aggaataacc
13801 gggatacaaa ccacgggtgg agaaccggac tccccacaac ctgaaaccgg gatataaacc
13861 acggctggag aaccggactc cgcacttaaa atgaaacaga aaccgggata aaaactacgg
13921 atggagaacc ggactccaca cattgagaca gaagaagttg tcagcccaga accccacacg
13981 agttttgcca ctgctaagct gtgaggcagt gcaggctggg acagccgacc tccaggttgc
14041 gaaaaacctg gtttctggga cctcccaccc cagagtaaaa agaacggagc ctccgctacc
14101 accctcccac gtggtggtag aaagacgggg tctagaggtt agaggagacc ctccagggaa
14161 caaatagtgg gaccatattg acgccaggga aagaccggag tggttctctg cttttcctcc
14221 agaggtctgt gagcacagtt tgctcaagaa taagcagacc tttggatgac aaacacaaaa
14281 ccactgggtc ggcatggcat ctccacctcc tcgcggtccg acctgggcat ccgaaggagg
14341 acgcacgtcc actcggatgg ctaagggaga gccacgagct cctcgacaga tcataatcag
14401 ccataccaca tttgtagagg ttttacttgc tttaaaaaac ctcccacacc tcccccctgaa
14461 cctgaaacat aaaatgaatg caattgttgt tgttaacttg tttattgcag cttataatgg
14521 ttacaaataa agcaatagca tcacaaattt cacaaataaa gcattttttt cactgcattc
14581 tagttgtggt ttgtccaaac tcatcaagat acgcgtacgc ggccccatgt tcgccttccg
14641 ccgcgtggag gaggatcaca gcaacaccga gctgggcatc gtggagtacc agcacgcctt
14701 caagaccccg gatgcagatg ccggtgaaga aagagtttaa acggccggcc gcggtcatag
14761 ctgtttcctg aacagatccc gggtggcatc cctgtgaccc ctccccagtg cctctcctgg
14821 ccctggaagt tgccactcca gtgcccacca gccttgtcct aataaaatta agttgcatca
14881 ttttgtctga ctaggtgtcc ttctataata ttatggggtg gaggggggtg gtatggagca
14941 aggggcaagt tgggaagaca acctgtaggg cctgcggggt ctattgggaa ccaagctgga
15001 gtgcagtggc acaatcttgg ctcactgcaa tctccgcctc ctgggttcaa gcgattctcc
15061 tgcctcagcc tcccgagttg ttgggattcc aggcatgcat gaccaggctc agctaatttt
15121 tgtttttttg gtagagacgg ggtttcacca tattggccag gctggtctcc aactcctaat
15181 ctcaggtgat ctacccacct tggcctccca aattgctggg attacaggcg tgaaccactg
15241 ctcccttccc tgtccttctg attttaaaat aactatacca gcaggaggac gtccagacac
15301 agcataggct acctggccat gcccaaccgg tgggacattt gagttgcttg cttggcactg
15361 tcctctcatg cgttgggtcc actcagtaga tgcctgttga attgggtacg cggccagctt
15421 ggctgtggaa tgtgtgtcag ttagggtgtg gaaagtcccc aggctcccca gcaggcagaa
15481 gtatgcaaag catgcatctc aattagtcag caaccaggtg tggaaagtcc ccaggctccc
15541 cagcaggcag aagtatgcaa agcatgcatc tcaattagtc agcaaccata gtcccgcccc
15601 taactccgcc catcccgccc ctaactccgc ccagttccgc ccattctccg ccccatggct
15661 gactaatttt ttttatttat gcagaggccg aggccgcctc ggcctctgag ctattccaga
15721 agtagtgagg aggctttttt ggaggcctag gcttttgcaa aaagctcccg ggagcttgta
15781 tatccatttt cggatctgat caagagacag gatgaggatc gtttcgcatg attgaacaag
15841 atggattgca cgcaggttct ccggccgctt gggtggagag gctattcggc tatgactggg
15901 cacaacagac aatcggctgc tctgatgccg ccgtgttccg gctgtcagcg caggggcgcc
15961 cggttctttt tgtcaagacc gacctgtccg gtgccctgaa tgaactgcag gacgaggcag
16021 cgcggctatc gtggctggcc acgacgggcg ttccttgcgc agctgtgctc gacgttgtca
16081 ctgaagcggg aagggactgg ctgctattgg gcgaagtgcc ggggcaggat ctcctgtcat
16141 ctcaccttgc tcctgccgag aaagtatcca tcatggctga tgcaatgcgg cggctgcata
16201 cgcttgatcc ggctacctgc ccattcgacc accaagcgaa acatcgcatc gagcgagcac
16261 gtactcggat ggaagccggt cttgtcgatc aggatgatct ggacgaagag catcaggggc
16321 tcgcgccagc cgaactgttc gccaggctca aggcgcgcat gcccgacggc gaggatctcg
16381 tcgtgaccca tggcgatgcc tgcttgccga atatcatggt ggaaaatggc cgcttttctg
16441 gattcatcga ctgtggccgg ctgggtgtgg ccgaccgcta tcaggacata gcgttggcta
16501 cccgtgatat tgctgaagag cttggcggcg aatgggctga ccgcttcctc gtgctttacg
16561 gtatcgccgc tcccgattcg cagcgcatcg ccttctatcg ccttcttgac gagttcttct
16621 gagcgggact ctggggttcg aaatgaccga ccaagcgacg cccaacctgc catcacgaga
16681 tttcgattcc accgccgcct tctatgaaag gttgggcttc ggaatcgttt tccgggacgc
16741 cggctggatg atcctccagc gcggggatct catgctggag ttcttcgccc accccaactt
16801 gtttattgca gcttataatg gttacaaata aagcaatagc atcacaaatt tcacaaataa
16861 agcatttttt tcactgcatt ctagttgtgg tttgtccaaa ctcatcaatg tatcttatca
16921 tgtctgtata ctggcttact atgttggcac tgatgagggt gtcagtgaag tgcttcatgt
16981 ggcaggagaa aaaaggctgc accggtgcgt cagcagaata tgtgatacag gatatattcc
17041 gcttcctcgc tcactgactc gctacgctcg gtcgttcgac tgcggcgagc ggaaatggct
17101 tacgaacggg gcggagattt cctggaagat gccaggaaga tacttaacag ggaagtgaga
17161 gggccgcggc aaagccgttt ttccataggc tccgcccccc tgacaagcat cacgaaatct
17221 gacgctcaaa tcagtggtgg cgaaacccga caggactata aagataccag gcgtttcccc
17281 tggcggctcc ctcgtgcgct ctcctgttcc tgcctttcgg tttaccggtg tcattccgct
17341 gttatggccg cgtttgtctc attccacgcc tgacactcag ttccgggtag gcagttcgct

```
17401 ccaagctgga ctgtatgcac gaacccccg ttcagtccga ccgctgcgcc ttatccggta
17461 actatcgtct tgagtccaac ccggaaagac atgcaaaagc accactggca gcagccactg
17521 gtaattgatt tagaggagtt agtcttgaag tcatgcgccg gttaaggcta aactgaaagg
17581 acaagttttg gtgactgcgc tcctccaagc cagttacctc ggttcaaaga gttggtagct
17641 cagagaacct tcgaaaaacc gccctgcaag gcggtttttt cgttttcaga gcaagagatt
17701 acgcgcagac caaaacgatc tcaagaagat catcttatta aggggtctga cgctcagtgg
17761 aacgaaaact cacgttaagg gattttggtc atgagattat caaaaaggat cttcacctag
17821 atccttttaa attaaaaatg aagttttaaa tcaatctaaa gtatatatga gtaaacttgg
17881 tctgacagtt accaatgctt aatcagtgag gcacctatct cagcgatctg tctatttcgt
17941 tcatccatag ttgcctgact ccccgtcgtg tagataacta cgatacggga gggcttacca
18001 tctggcccca gtgctgcaat gataccgcga gacccacgct caccggctcc agatttatca
18061 gcaataaacc agccagccgg aagggccgag cgcagaagtg gtcctgcaac tttatccgcc
18121 tccatccagt ctattaattg ttgccgggaa gctagagtaa gtagttcgcc agttaatagt
18181 ttgcgcaacg ttgttgccat tgctgcaggc atcgtggtgt cacgctcgtc gtttggtatg
18241 gcttcattca gctccggttc ccaacgatca aggcgagtta catgatcccc catgttgtgc
18301 aaaaaagcgg ttagctcctt cggtcctccg atcgttgtca gaagtaagtt ggccgcagtg
18361 ttatcactca tggttatggc agcactgcat aattctctta ctgtcatgcc atccgtaaga
18421 tgcttttctg tgactggtga gtactcaacc aagtcattct gagaatagtg tatgcggcga
18481 ccgagttgct cttgcccggc gtcaacacgg gataataccg cgccacatag cagaacttta
18541 aaagtgctca tcattggaaa acgttcttcg gggcgaaaac tctcaaggat cttaccgctg
18601 ttgagatcca gttcgatgta acccactcgt gcacccaact gatcttcagc atcttttact
18661 ttcaccagcg tttctgggtg agcaaaaaca ggaaggcaaa atgccgcaaa aaagggaata
18721 agggcgacac ggaaatgttg aatactcata ctcttccttt ttcaatatta ttgaagcatt
18781 tatcagggtt attgtctcat gagcggatac atatttgaat gtatttagaa aaataaacaa
18841 ataggggttc cgcgcacatt tccccgaaaa gtgccacctg acgtgtcgac gcggccgcac
18901 attgattatt gactagttat taatagtaat caattacggg gtcattagtt catagcccat
18961 atatggagtt ccgcgttaca taacttacgg taaatggccc gcctggctga ccgcccaacg
19021 acccccgccc attgacgtca ataatgacgt atgttcccat agtaacgcca atagggactt
19081 tccattgacg tcaatgggtg gagtatttac ggtaaactgc ccacttggca gtacatcaag
19141 tgtatcatat gccaagtccg ccccctattg acgtcaatga cggtaaatgg cccgcctggc
19201 attatgccca gtacatgacc ttacgggact ttcctacttg gcagtacatc tacgtattag
19261 tcatcgctat taccatggtg atgcggtttt ggcagtacac caatgggcgt ggatagcggt
19321 ttgactcacg gggatttcca agtctccacc ccattgacgt caatgggagt ttgttttggc
19381 accaaaatca acgggacttt ccaaaatgtc gtaataaccc cgccccgttg acgcaaatgg
19441 gcggtaggcg tgtacggtgg gaggtctata taagcagagc tcgtttagtg aaccg
//
```

Figure S3. The complete nucleotide sequence of the molecular infectious clone pYFrep/GAG-POL/Pac.

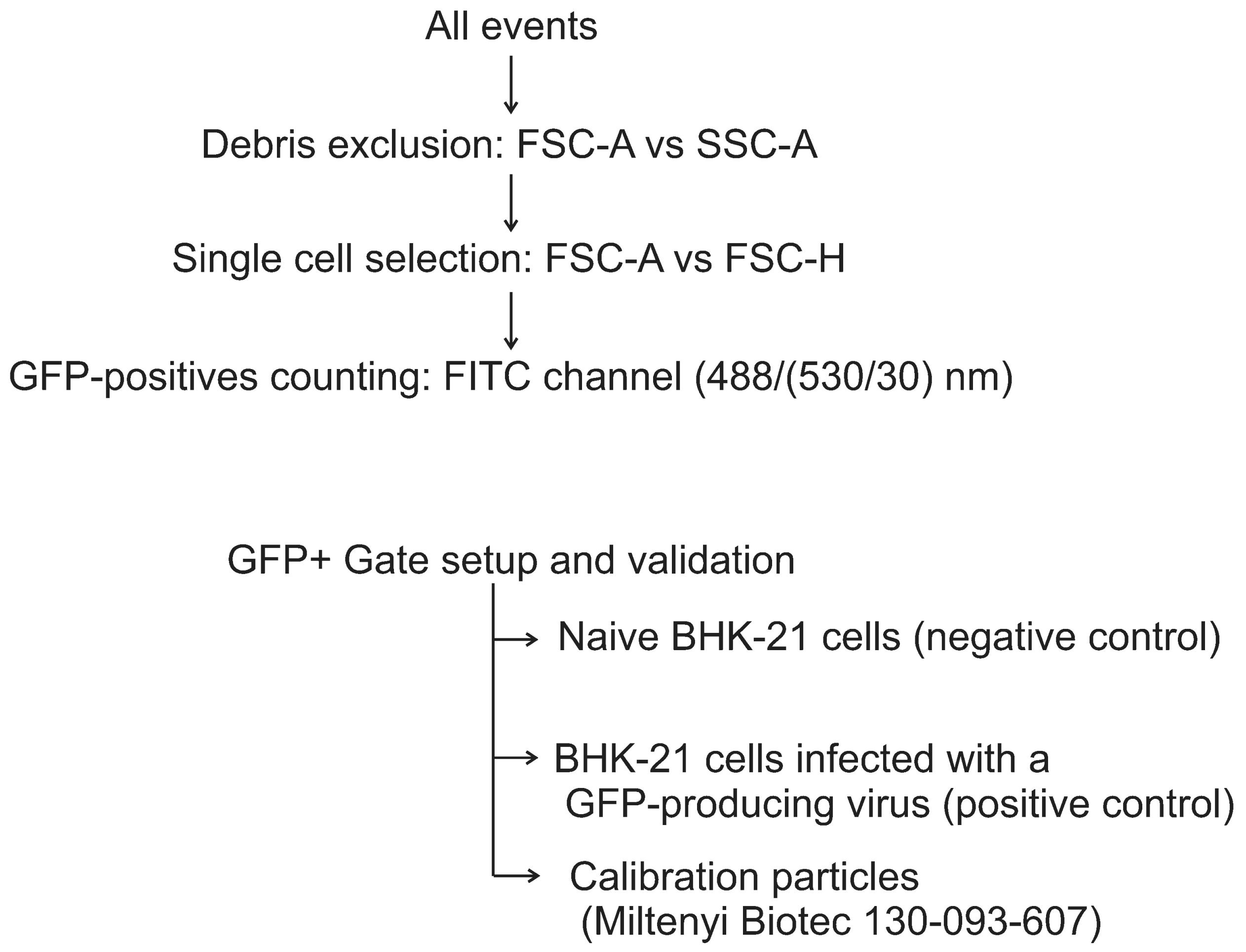

Figure S4. Flow cytometry gating strategy for counting GFP-positive cells.
The proportion (%) of GFP-positive cells was measured during titration of LV particles encoding GFP using the sequential gating strategy illustrated.

Table S1. Primers used for sequencing the Gag-Pol insert in the replicon.

Primer	Orientation	Sequence 5′-3′	Position, nt [1]	Region in the replicon
F1	Forward	GCGACGTGGAGGAAAACCCTG	(-26) – (-6)	T2A peptide preceding Gag
F2	Forward	CTATTGCACCAGGCCAGATGA	665 – 685	Gag
F3	Forward	GAGAGCTTCAGGTTTGGGGAA	1381 – 1401	Gag
F4	Forward	GTACTGGATGTGGGCGATGCA	2082 – 2102	Pol
F5	Forward	AATACAGAAGCAGGGGCAAGG	2744 – 2764	Pol
F6	Forward	GATGGAATAGATAAGGCCCAA	3447 – 3467	Pol
R1	Reverse	GGGCGGAATTCTTAATCCTCA	4298 – 4318	Junction Pol/IRES
R2	Reverse	GGGCTACAGTCTACTTGTCCA	3593 – 3613	Pol
R3	Reverse	CTTTCCCCATATTACTATGCT	2895 – 2915	Pol
R4	Reverse	CTGTACTGTCCATTTATCAGG	2466 – 2486	Pol
R5	Reverse	TAAAGTGCAGCCAATCTGAGT	1734 – 1754	Pol
R6	Reverse	ATCTGGGTTCGCATTTTGGAC	967 – 987	Gag

Comment [1] The adenine in the starting ATG codon of the GAG gene is designated +1.

Table S2. Representative data used to compute the limit of detection (LOD) for p24 ELISA.

Well [1]	OD_{450}	p24 (pg/mL) [2]
1	0.015	1.48
2	0.014	0.74
3	0.016	2.23
4	0.017	2.97
5	0.019	4.45
6	0.011	-1.48
7	0.017	2.97
8	0.016	2.23
9	0.018	3.71
10	0.010	-2.23
Mean		1.61
SD		2.16
LOD (mean + 3 × SD)		8.09

Comment: [1] Ten wells were measured for LOD. Conditioned medium from naïve C8166 cells was used for the ten samples. [2] Negative p24 values for some wells arise because their OD_{450} readings fall below the ELISA kit's blank, indicating lower background.

Standard curve equation (OD_{450} from p24(pg/ml)): $y = 0.001348x + 0.013$

Reverse equation p24(pg/ml) = (OD_{450} − 0.013) / 0.001348

Computed LOD = 8.09 pg/mL

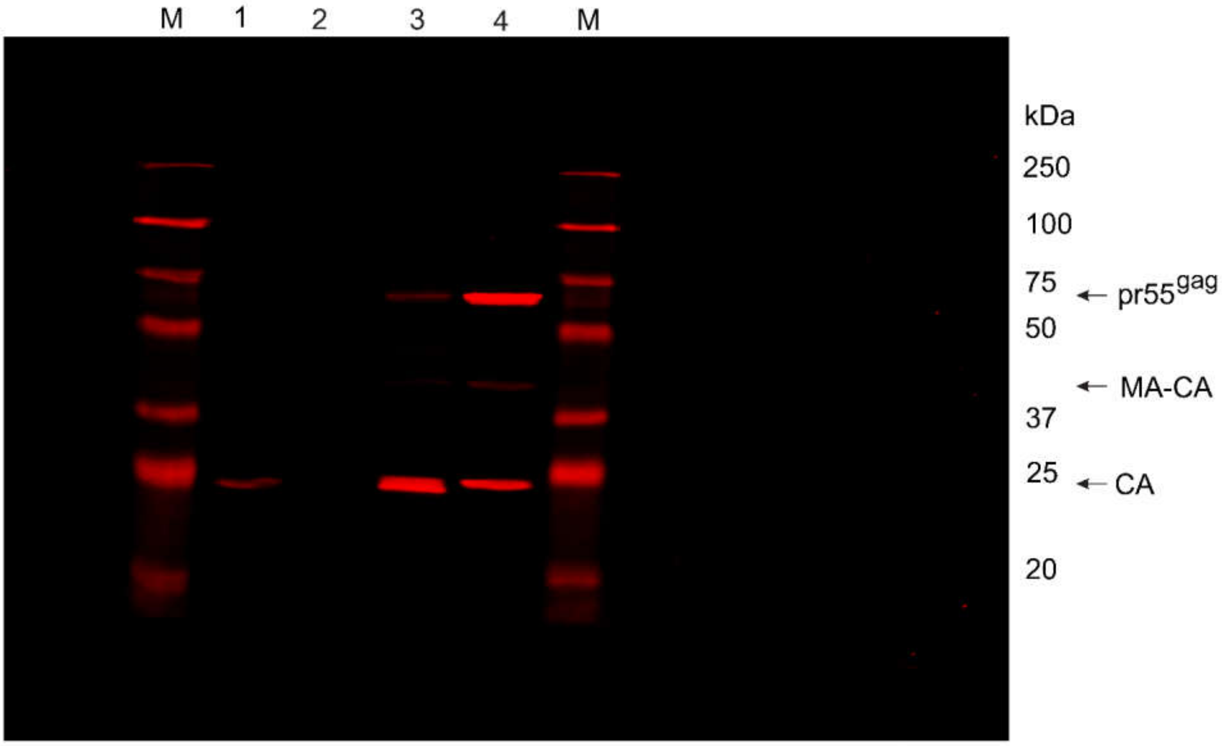

Figure S5. Full membrane image of Western blot analysis of Gag processing in HEK293FT cells. Lanes: 1, recombinant p24 protein (Thermo Scientific Cat. RP-4916); 2, lysate of naïve HEK293FT cells; 3, lysate of cells carrying the replicon YFrep/GFP-GAG-POL (with wild-type HIV-1 protease); 4, lysate of cells carrying the replicon YFrep/GFP-GAG-POL* (has the T26S mutation in the protease). M, Bio-Rad Precision Plus Protein Standard (Cat. #1610373); molecular masses (kDa) are indicated on the right. The membrane was stained with a primary anti-p24 antibody followed by an IRDye 680RD-labeled secondary antibody and scanned on a LI-COR Odyssey system in the 680 nm channel.

a

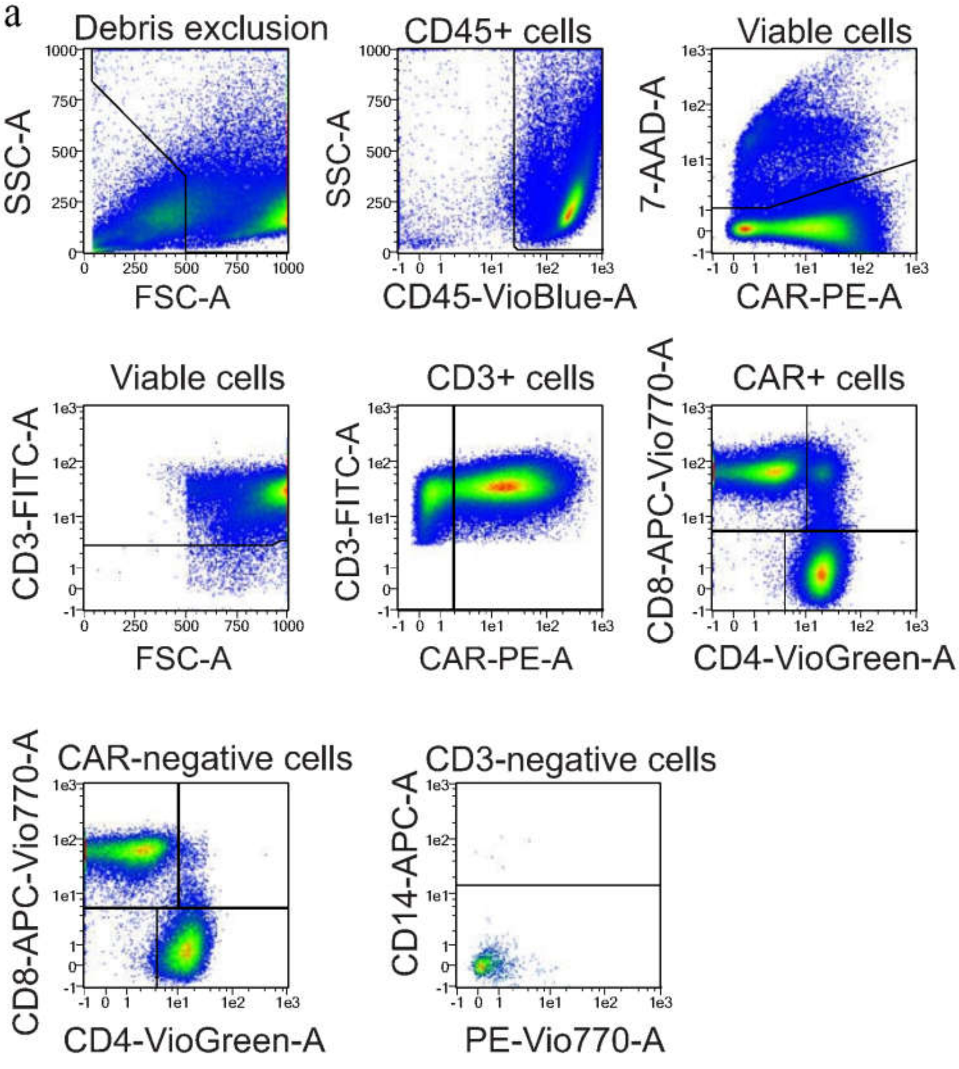

b

Cell type	Defined population	Cells / mL	Count	Percent
Sample	all acquired events	8.86E+06	257781	
Debris exclusion	FSC small events excluded	7.73E+06	225013	87.29
CD45+ cells	FSC small events excluded and CD45+	7.67E+06	223296	99.24
Viable CD45+ cells	CD45+ 7-AAD-	6.83E+06	198661	88.97
CD3+ cells	Viable CD3+	6.78E+06	197280	99.30
CAR+ cells	Viable CD3+ CAR+	5.25E+06	152710	77.41
CAR+ CD4+ cells	Viable CD3+ CAR+ CD4+ CD8-	2.21E+06	64270	42.09
CAR+ CD8+ cells	Viable CD3+ CAR+ CD4- CD8+	2.64E+06	76978	50.41
CAR+ CD4+ CD8+ cells	Viable CD3+ CAR+ CD4+ CD8+	3.88E+05	11307	7.40
CAR+ CD4- CD8- cells	Viable CD3+ CAR+ CD4- CD8-	5.33E+03	155	0.10
CAR- cells	Viable CD3+ CAR-	1.53E+06	44570	22.59
CAR- CD4+ cells	Viable CD3+ CAR- CD4+ CD8-	6.91E+05	20107	45.11
CAR- CD8+ cells	Viable CD3+ CAR- CD4- CD8+	7.90E+05	22991	51.58
CAR- CD4+ CD8+ cells	Viable CD3+ CAR- CD4+ CD8+	3.34E+04	972	2.18
CAR- CD4- CD8- cells	Viable CD3+ CAR- CD4- CD8-	1.72E+04	500	1.12
CD3- cells	Viable CD3-	4.74E+04	1381	0.70
Monocytes	Viable CD3- CD14+ among CD45+ 7-AAD-	1.72E+02	5	0.00

				Percent
CAR+ cells in viable CD3+	CD3+ CAR+ in CD45+ 7-AAD- CD3+	5.25E+06	152710	77.41
CAR+ cells in viable cells	CD3+ CAR+ in CD45+ 7-AAD-	5.25E+06	152710	76.87

Figure S6. Characterization of cell types in the CAR-T cell product obtained using the lentiviral vector LV/CAR packaged in replicon cell pools (HEK293FT-GP).

(**a**) Representative flow cytometry scatter plots showing the distribution of cell populations by surface marker expression. The gates used to isolate cell populations are shown. Markers are indicated on the axes.
(**b**) Summary table of cell type representation in the CAR-T cell product: cell type, gating strategy, event count, and percentage (calculated relative to all viable cells or to viable $CD3^+$ T cells).
